# Inter-segmental coordination patterns in Parkinson’s disease are particularly disturbed during preferred walking speed: a data-driven network approach

**DOI:** 10.1186/s12984-025-01835-1

**Published:** 2025-12-12

**Authors:** Karolina Saegner, Robbin Romijnders, Inga Ruff, Julius Welzel, Clint Hansen, Elke Warmerdam, Pedro Conceição, Walter Maetzler

**Affiliations:** 1 Department of Neurology, University Hospital Schleswig-Holstein, Campus Kiel and Kiel University, Arnold-Heller Str. 3, 24105 Kiel, Germany; 2 Department of Psychology, Carl von Ossietzky Universität Oldenburg, Oldenburg, Germany; 3 Biomedical signals and systems, Faculty of Electrical Engineering, Mathematics and Computer Science, University of Twente, Enschede, The Netherlands; 4 Institute of Computer Science, Dependable Systems, Kiel University, Kiel, Germany

**Keywords:** Neurogeriatrics, Gait analysis, Kinectome, Network, Graphs

## Abstract

**Background:**

Human gait involves complex coordination between musculoskeletal segments. This coordination is disturbed in Parkinson’s disease (PD) and likely influenced by different walking speeds.

**Objectives:**

To investigate inter-segmental coordination during different walking speeds in people with PD (PwPD) using an unconstrained and data-driven network theory approach.

**Methods:**

Twenty-nine PwPD and 29 controls walked at preferred, fast and slow speeds. Data was collected using optical motion capture. Body segment accelerations were correlated pairwise to build kinectomes for each speed and movement direction. Anatomical body segments were defined as nodes and their co-accelerations as edges to build network graphs. The kinectomes and maximum-weighted graph patterns were compared between groups.

**Results:**

Permutation testing revealed no significant kinectome differences between groups across speeds or directions. Coordination deficits in the PD group were observed predominantly at preferred walking speed (162 significantly different graph patterns) in anteroposterior and mediolateral directions. At fast walking speed, 4 significantly different graph patterns were found in anteroposterior and vertical directions. Slow walking speed showed 1 significantly different pattern in mediolateral direction.

**Conclusions:**

PD affects inter-segmental coordination, becoming most apparent at preferred walking speed. This is surprising and highly relevant, as it is the most common gait condition in real life. ‘Non-preferred’ walking speeds in PD exhibit more control-like patterns, which could inform future treatment studies. The direction-specific coordination deficits could provide novel insights into patho- and compensatory mechanisms in PD gait.

*Trial registration* The study is registered in the German Clinical Trials Register (DRKS00022998, registered on 04 Sep 2020).

**Supplementary Information:**

The online version contains supplementary material available at 10.1186/s12984-025-01835-1.

## Introduction

Parkinson’s disease (PD) is the second most common [1] and the fastest growing [2] neurodegenerative disease worldwide. Typical motor symptoms include bradykinesia, resting tremor, rigidity, freezing of gait, and unstable posture [3, 4]. Furthermore, people with PD (PwPD) often have gait disturbances, such as shuffling gait, increased difficulty lifting feet from the ground, decreased stride length and velocity [4, 5], impaired foot placement [6], and reduced range of walking speed [7]. Importantly, these disturbances are associated with activity limitations during daily life [8].

Typically gait of PwPD is evaluated with parameters that can be extracted from the lower extremities and the lower back, including velocity, stride and step length and width, cadence, gait phase duration, and variability and asymmetry of these metrics, as well as the range of motion of the joints of the lower extremities [9, 10]. However, for an overall view of the entire human body as an integrated system it is important to capture many simultaneous interactions arising from coordinated work of different musculoskeletal segments. This can be analysed with information coming from more than only one area of the body [11] and, for complex analyses, using the network theory [12]. The latter offers a framework for investigating complex systems, consisting of many interconnected elements, and how they interact with each other. This approach allows insights into the overall behaviour of the whole system [12, 13].

Troisi Lopez et al. [14] have used principles from network theory to explore human locomotion. For that purpose, they have established a *kinectome*, where human gait is represented as a matrix of acceleration correlations between the body segments. The kinectome was interpreted as a network, where the nodes corresponded to body segments, and edges were defined by the correlation of acceleration signals between the segments [14, 15]. Using a variety of analysis methods, such as modularity and various topological measures, they were able to distinguish between PwPD and controls and to predict clinical symptoms in PD [14]. More specifically, they showed a reduced joint coordination in PwPD compared to controls [16], and were able to demonstrate an effect of dopaminergic treatment on this coordination [17].

However, to our best knowledge, how the full body is involved in walking speed-related deficits has not been adequately investigated to date. Previous research has demonstrated that PwPD exhibit both upper and lower limb asymmetry during gait [18, 19] and that rigidity contributes to reduced forward limb propulsion in PD, thus negatively affecting walking speed and step length [20]. Furthermore, PwPD have difficulties in modulating their walking speed [7] and adapting to changes in walking speed [21]. The interconnected nature of segmental coordination [22] means that deficits in one body region can affect coordination patterns throughout the entire kinematic chain [23], necessitating a comprehensive full-body analysis approach. To address the questions about the effect of different walking speed on inter-segmental coordination, which is defined as a relationship between different body segments during movement, we used the aforementioned network theory [14] and applied it to a dataset produced in our lab [11]. We hypothesized that the inter-segmental coordination during walking differs between PwPD and healthy controls in lower and upper parts of the body, with the differences depending on different walking speeds.

## Methods

### Ethics approval and consent to participate

The study was approved by the ethics committee of the Medical Faculty of Kiel University (D438/18) and was conducted in accordance with the principles of the Declaration of Helsinki. The study is registered in the German Clinical Trials Register (DRKS00022998, registered on 04 Sep 2020). All study participants have read and signed an informed consent prior to the study.

### Participants, in-and exclusion criteria

Participants aged 18 years or older who were able to walk independently without the use of walking aids were eligible for inclusion. Individuals were excluded if they scored below 15 on the Montreal Cognitive Assessment or if they had other movement disorders known to impact mobility, based on evaluation by a movement disorder specialist (WM). For full details regarding inclusion and exclusion criteria, see Warmerdam et al. [11].

A total of 58 participants were included in the present analysis. This comprised 29 PwPD (11 females), i.e., the PD group, who performed walking tasks during their self-reported optimal “ON” medication state, assessed between 30 and 120 min after oral intake of levodopa. The control group consisted of 29 age-matched healthy adults (14 females), with no comorbidities affecting mobility, as confirmed by the same movement disorder specialist. Demographic and clinical characteristics are summarized in Table 1.

**Table 1:** Demographic and clinical parameters of the participating groups

Group	Controls	PD
Total *N* (males/females)	29 (15/14)	29 (18/11)
Age (years)	67 ± 12	67 ± 10
Height [cm]	176 ± 10	174 ± 9
Weight [kg]	79 ± 16	82 ± 18
BMI [kg/m^2^]	26 ± 5	27 ± 5
Disease duration (years)	-	9 ± 6
Hoehn & Yahr (1–5)	-	3 ± 1
Medication dose (LEDD)	-	713 ± 301
MDS-UPDRS III	4 ± 4	29 ± 21

The values are displayed as mean ± standard deviation

*PD* Parkinson’sdisease, *BMI* Body Mass Index, *LEDD* Levodopa equivalent daily dose, *MDS-UPDRS III* Motor part of the Movement Disorder Society Unified Parkinson’s Disease Rating Scale

### Determining the most and least affected sides

The most and least affected upper extremities and lower extremities were determined based on the motor part of the Movement Disorder Society Unified Parkinson’s Disease Rating Scale (MDS-UPDRS III) [24] scores, namely the sum of items 3.3 (rigidity), 3.4 (finger tapping), 3.5 (hand movements), and 3.6 (pronation and supination of the hand) for left and right upper extremity, and the sum of 3.3 (rigidity), 3.7 (toe tapping), and 3.8 (leg agility) for left and right lower extremity. A difference of ≥ 1 point was considered as having lateralization [25]. If the MDS-UPDRS III scores between the sides were even, as well as for the control group, the non-dominant hand was taken as the most affected side due to lower motor performance on the non-dominant side [26]. For participants missing the handedness data (9 PD and 6 controls), the left hand was taken as non-dominant due to handedness distribution in the general population, where around 90% of all people are right-handed [27].

### Equipment, data acquisition and data dimensionality reduction

**Fig. 1:**
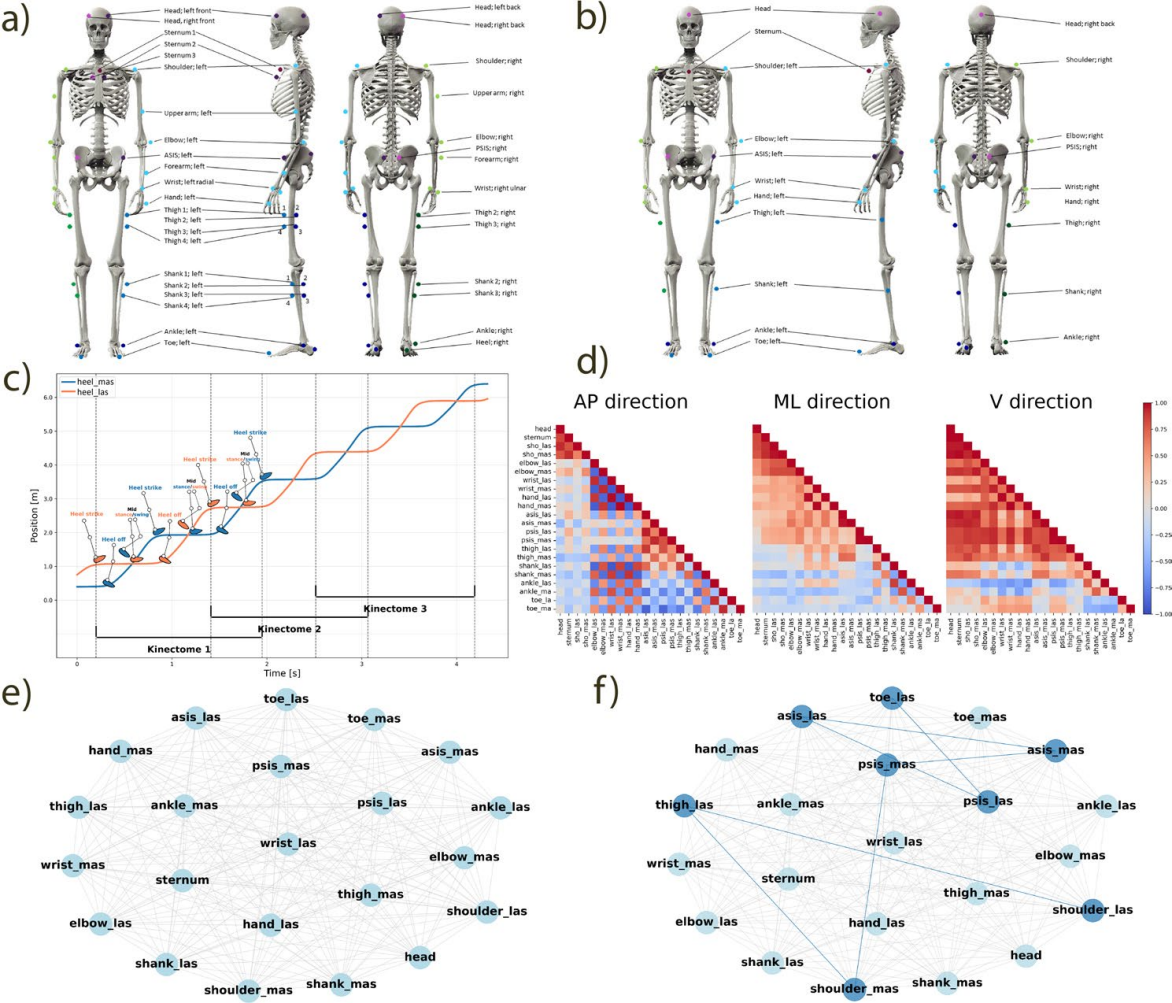
Scheme of the kinectome analysis. **a** Original marker positions; **b** Marker positions after reduction; **c** Position data of the most and least affected heels; data cutting into one full left and one full right gait cycles = one kinectome; **d** Example kinectomes in anteroposterior, mediolateral, and vertical directions; **e** Representation of the kinectome as a graph; **f** Strongest pattern in the graph (dark blue). a and b were adapted from Warmerdam et al. (2021) with permission [11]

All the motor tasks were recorded using a twelve-camera optical motion capture system recording with 200 Hz (Qualisys AB, Göteborg, Sweden). A total of 47 reflective markers were placed on the body (Fig. 1a). In addition, all the trials were videotaped by two cameras (GoPro Inc., Hero Session, San Mateo, CA, USA). Full details on the experimental protocol can be found in Warmerdam et al. [11]. For the present study, the four head markers were calculated into one midpoint, the 3-marker cluster on the sternum was calculated into one midpoint, the 4-marker clusters on the thighs and shanks were calculated into one midpoint per segment and per side, and the upper arm, forearm, and heel marker locations were excluded from further data analysis. The resulting 22 marker data (Fig. 1b), where each marker represented a body segment, were taken for further analysis.

### Motor assessment

All study participants were instructed to walk a straight path along a 5 m long and 1 m wide walkway. There were three walking trials, namely at preferred (“Please walk at your normal walking speed”), fast (“Please walk as fast as possible, without running, falling or feeling unsafe”), and slow (“Please walk half of your normal walking speed”) speeds. The walking speed and the differences between the trials can be found Supplementary Fig. 1. The beginning and end of the walkway were marked by cones with reflective markers. The participants were asked to start walking approximately 2 m before the start cones and finish walking approximately 2 m behind the end cones to make sure that steady state walking was measured within the 5 m gait trial and any variation in gait appearing due to gait initiation and termination [7, 28] is excluded from the data acquisition.

### Marker data pre-processing

Marker data were prepared in a format following the Brain Imaging Data Structure (BIDS) [29, 30] and were pre-processed using Python (Python Software Foundation. Python Language Reference, version 3.11.2). The walking data were trimmed to only contain the samples collected between the start and end cones. These data were then cut so each gait acquisition contains one full left and one full right gait cycle (Fig. 1c). Missing values were linearly interpolated using the fill_missing_samples function from Kinetics Toolkit [31] with the threshold of 271 samples (1.35 s). The remaining data were filtered using a 2nd order Butterworth filter with the cut-off frequency of 6 Hz. Next, the data were rotated using Principal Component Analysis to align the X direction of the markers with the X direction of the laboratory’s coordinate system, so the X direction becomes the anteroposterior (AP) direction of walking. This rotation was performed individually for each participant to ensure accurate alignment, particularly since the walking direction may not perfectly align with the laboratory’s global axes, especially in people with non-normal gait characteristics. Lastly, the data, originally containing positions, was differentiated twice to get the acceleration of the markers.

### Building the kinectome

Time series containing acceleration data of each marker during each gait acquisition in the anteroposterior (AP) (the forward-backward direction aligned with the primary direction of progression during walking [32]) mediolateral (ML) (the side-to-side direction perpendicular to the walking direction), and vertical (V) (the superior-inferior direction aligned with the gravity vector) directions were first normalised to 500 samples, and then were used to build person, trial and gait acquisition specific kinectomes. The kinectomes, each containing one full gait cycle from each body side (> 3 available for each trial), were built in each movement direction separately by correlating the acceleration data from each marker with that from every other marker, resulting in a mirrored 22✕22✕3 correlation matrix (where 22 is the number of body segments, and 3 is the number of movement directions, Fig. 1d). This matrix will be referred to as the kinectome from now on [14]. In this paper we present the results obtained using Pearson’s correlation.

Then for each person an average kinectome was built by averaging the kinectomes from multiple gait kinectomes obtained from the respective trial in a respective movement direction (e.g., averaging kinectomes one, two, and three in Fig. 1c). For each person standard deviation of the kinectomes was obtained by calculating the standard deviation between all gait kinectomes (Fig. 1c) for each trial and movement direction separately. This resulted in each person having one person-specific average and one person-specific standard deviation kinectome per trial and direction (total of 9 average and 9 standard deviation kinectomes per person).

### Group kinectome analysis

The individual average and standard deviation kinectomes were used to build group-specific average and standard deviation kinectomes, respectively, for each walking speed and each movement direction separately for the control and PD groups, by averaging all individual kinectomes between the people within the groups. The latter kinectomes were used for permutation analysis.

### Representing the kinectome as a graph

Each person-specific average kinectome was represented as a graph (Fig. 1e) for further analysis. The body segments were represented as nodes, and the acceleration correlations between them as edges between the nodes. These graphs were non-directional, i.e., there was no particular direction of the edges between the nodes; and complete, i.e., each node was connected with each other node, based on the assumption that motor coordination involves relationships between anatomically distant joints (e.g. contralateral limb swing during gait) [22]. Network analysis was done using NetworkX package (Version 3.4.2) [33] for Python.

Marker trajectories were analyzed in the global coordinate system. This approach differs from traditional joint kinematics and is appropriate for kinectome analysis, as it preserves whole-body coordination patterns and treats each body segment as an independentnode within the motor control network.

### Patterns

A pattern of a graph refers to a specific arrangement of its nodes and edges (Fig. 1f). Here we analysed path patterns, i.e., sequences of connected nodes and edges. In this paper, a path refers to a sequence of nodes connected by edges, and a pattern is a path represented in the kinematic data. We have looked at the maximum-weighted patterns in the respective person-specific average kinectome that started from a given node and had a fixed length. This approach provided an unconstrained and data-driven view on respective comparisons. These comparisons were done on three arbitrarily defined levels, namely local (2–6 body segments in the pattern), referring to simple coordination networks, multi-segmental (7–14 body segments) – more complex networks, and whole-body (15 + body segments) – global coordination networks. The maximum-weighted patterns were chosen for the analysis because they provide the baseline for understanding coordination differences between the groups.

The algorithm src.graph_utils.kinectome2pattern.strongest_pattern_subgraph (available on [34]) starts at a given node and constructs a path by iteratively selecting edges with the highest absolute weight while avoiding node repetition. This ensures that each subsequent step in the path represents the strongest local connection available from the current position, creating a path that follows the most prominent signal through the network structure. Note that while the starting node is fixed, there is no predetermined target node—only the path length is constrained.

Since there are 22 bone segments taken for the analysis, the algorithm looped over each body segment and took it as a starting node. Pattern lengths ranging from 2 to 20 nodes were considered. First, the strongest pattern for each individual was found. Then, within each group, patterns of the same length and starting node were averaged to determine the strongest group-specific pattern. Finally, the values of this pattern were then taken from each individual in both groups and compared statistically.

This strategy enabled an exploratory analysis of emerging patterns; rather than specifying a particular pattern, it allowed the algorithm to first identify the strongest pattern and then assess the differences between these patterns across groups.

### Statistics

Statistical analysis was done using SciPy [35] for Python and JASP (JASP Team (2023). JASP (Version 0.18)). The characteristics of the groups are presented as mean ± standard deviation. Normality distribution was checked using the Shapiro-Wilk test. Outliers were removed using the Z-score method (removing values three standard deviations above or below the group mean) for normally distributed data, or by calculating the interquartile range and removing values 1.5 times greater or less than the interquartile range for non-normally distributed data.

Chi-square test was used to compare the gender distribution between the groups. To compare all other variables between the groups, t-test and Mann-Whitney U tests were used for normally and non-normally distributed data, respectively.

The group-specific average and standard deviation kinectomes were compared through permutation testing, by randomly shuffling the control group kinectomes 5000 times. Spearman’s rho was used to to quantify the correlation between group kinectomes and generate the distribution of the correlations expected by chance alone [36, 37]. To assess the stability of the kinectomes across sample sizes, we performed bootstrap resampling (*n* = 5000) at varying percentages (10–90%) of the original dataset. For each bootstrap iteration, kinectomes for each walking speed and movement direction were computed using the subset of the full sample, and permutation testing was applied to these subsets to build a bootstrap distribution of correlations.

There were two pattern analyses done, namely strongest patterns found in the control group were compared to the same patterns in the PD group, and strongest patterns found in the PD group were compared to those in the control group. The patterns derived from the control group are presented in the result section, and the results comparing PD group’s patterns can be found in Supplementary Table 1. Patterns with an overlap of 80% as determined by Jaccard index [38] were seen as the same pattern. Higher values of the pattern indicate stronger correlation between the body segments of that particular pattern. The patterns between the groups were compared using t-test or Mann-Whitney U test for normally and non-normally distributed data, respectively. The effect size was calculated using Cohen’s d. Effect size measure was interpreted as small, medium, or large if the effect size was 0.2 < x < 0.5, 0.5 < x < 0.8, and x ≥ 0.8, respectively [39]. To adjust for multiple comparisons when comparing the patterns of the same length, but different starting nodes, a Bonferroni correction was used (*n* = 22). All comparisons were made between the groups within the same walking speed and same movement direction.

## Results

There were no significant differences between the groups in gender distribution, age, height, weight, or body mass index (*p* > .05). The MDS-UPDRS III scores differed between the control and PD groups, with the latter group having higher scores. No freezing episodes were observed in the PD group, as revealed by the video data. Details are shown in Table 1.

### Comparing the group-specific kinectomes

Average and standard deviation kinectomes’ comparisons between the PD and control groups using permutation testing did not reveal any significant differences. The correlations between the average and between the standard deviation kinectomes are unlikely to have occurred by chance, and the observed correlation is stronger than what could be expected by random chance. All 18 (2 kinectome types × 3 walking conditions × 3 movement directions) correlation coefficients are reported in Table 2.

**Table 2:** Spearman’s Rho values between the group-specific kinectomes for preferred, fast, and slow walking speeds

Spearman’s rho	Preferred			Fast			Slow			
	AP	ML	V	AP	ML	V	AP	ML	V	
Average kinectomes	0.99	0.93	0.97	0.98	0.91	0.92	0.98	0.94	0.98	
Standard deviation kinectomes	0.73	0.63	0.83	0.88	0.61	0.66	0.88	0.74	0.80	

Average kinectomes were built by averaging the gait-specific kinectomes for each participant per walking speed and movement direction. Standard deviation kinectomes were built by calculating the standard deviation between the gait-specific kinectomes for each participant per walking speed and movement direction

**Fig. 2:**
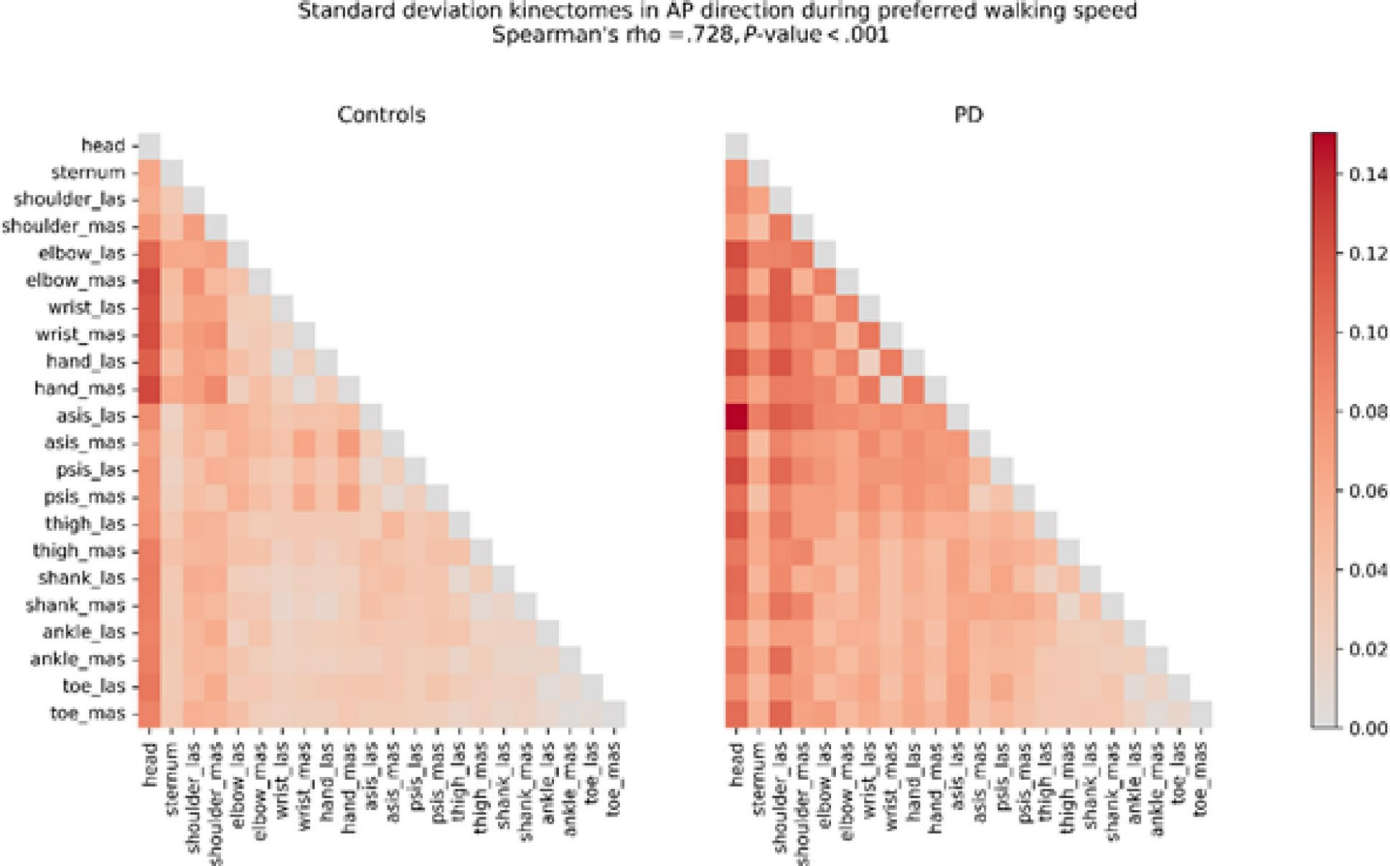
Standard deviation kinectomes of Control and PD groups obtained from preferred walking speed trials, AP direction. Warmer colours indicate higher standard deviation. The *P*-value is obtained from permutation testing. *AP* anteroposterior direction, *asis* anterior superior iliac spine, *las* least affected side, *mas* most affected side, *PD* Parkinsons’s disease group, *psis* posterior superior iliac spine

Spearman’s rho values between control and PD standard deviation kinectomes were consistently lower than those for the average kinectomes across all walking speeds and directions. A representative comparison of a standard deviation kinectomes from preferred walking trials in AP direction can be found in Fig. 2. Moreover, the standard deviation values were consistently higher in the PD group than in the control group. These findings indicate that while the overall structure of the inter-segmental coordination is similar between the groups, the variability in this inter-segmental coordination is higher in PD group than in controls.

#### Bootstrapping

Bootstrap analysis demonstrated high correlation stability across all conditions. The correlations approached asymptotic values by 40–60% sample size for average and 80–90% for standard deviation kinectomes. Using 80% of the original sample for the subset size, the observed correlations exceeded bootstrap means (Supplementary Figs. 3 and 4).

### Graph patterns

Pattern analysis revealed distinct differences in movement coordination patterns between PD and control groups across movements in AP, ML, and V directions mainly during preferred and fast walking, while slow walking had only one significantly different pattern. Full patterns can be found in Supplementary Table 2.

The results consistently show altered inter-segmental coordination in the PD group across various organizational levels (local, multi-segmental, and whole-body). Importantly, the manifestation and characteristics of these deficits varied with walking speed.

#### Preferred walking speed

**Fig. 3:**
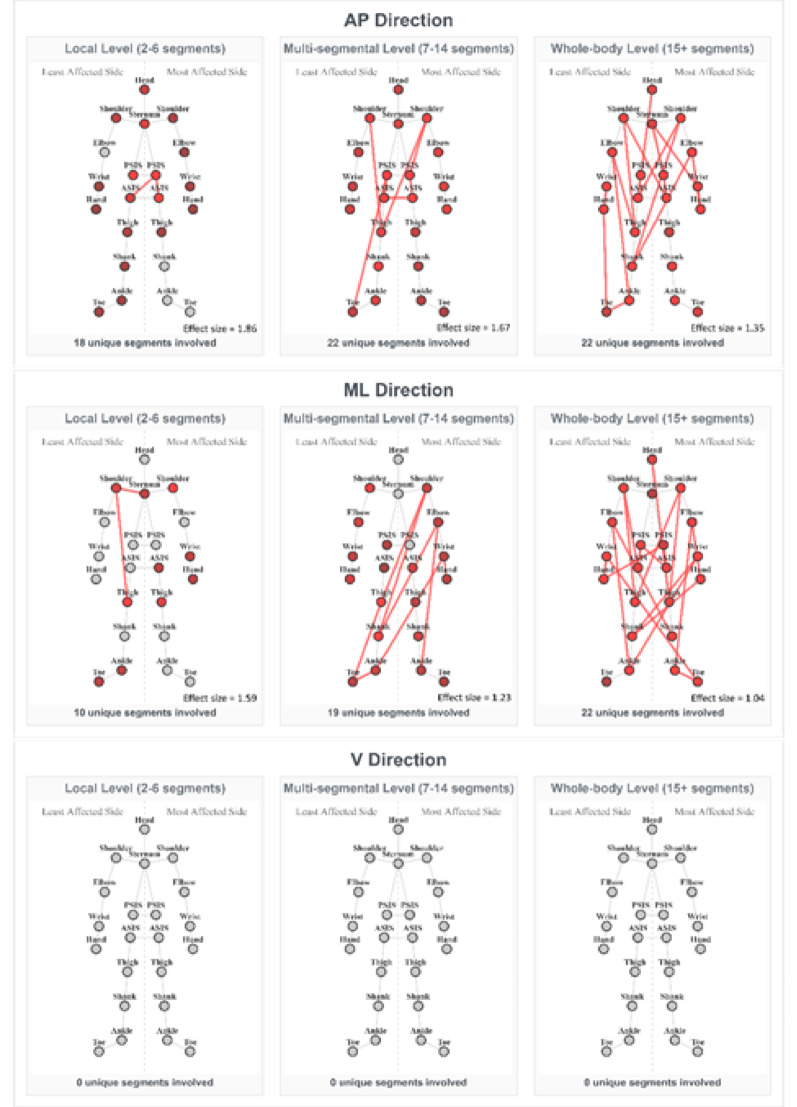
Significantly different coordination patterns between PD and controls at preferred walking speed. The patterns with largest effect sizes are shown. Connected nodes indicate the strongest coordination deficit in pwPD; coloured but unconnected nodes appear in other significant patterns. Red indicates weaker inter-segmental coordination in the PD group than in the control group. The colour intensity reflects the frequency each node appeared in all significant patterns for the given speed, direction, and segmental level (more vivid - higher frequency). ASIS – anterior superior iliac spine; PSIS – posterior superior iliac spine

At preferred walking speed, the inter-segmental coordination deficits of the PD group were most apparent (Fig. 3). The reduction in coordination was consistent and multi-level in both the AP and ML directions. In the following, the main findings are summarized:

- *AP direction* the PD group showed weaker coordination than controls across all organizational levels, with the most significantly different patterns observed in this speed and direction (*n* = 146):
  - On a local level (*n* = 26), patterns mostly involving axial segments (head, trunk, pelvis), both upper limbs, and the less affected lower limb, were weaker in the PD group (effect sizes: 0.53–1.86, mean = 0.8). This suggests deficits in basic postural coordination.
  - On a multi-segmental level (*n* = 80), patterns involving multiple body regions and including contralateral limb movement were weaker in the PD group (effect sizes: 0.53–1.67, mean = 0.78). These findings suggest PD-related deficits in simultaneous multi-segmental coordination during walking.
  - On a whole-body level (*n* = 40), patterns involving most body segments showed the most pronounced deficits in the PD group (effect sizes: 0.53–1.35, mean = 0.7). This suggests that PwPD cannot effectively coordinate whole-body inter-segmental movements during walking.
- *ML direction* Weaker inter-segmental coordination was observed across all organizational levels in the PD group compared to controls, although notably fewer patterns were significantly different (*n* = 16) compared to the AP direction.
  - On a local level (*n* = 5), patterns involving sternum, the less affected shoulder and thigh, as well as between the most affected upper limb and several segments in both most and least affected lower limbs, were weaker in the PD group (effect sizes: 0.54–1.59, mean = 1.33).
  - On a multi-segmental level (*n* = 7), patterns primarily including the pelvis, and upper and lower limbs, were weaker in the PD group (effect sizes: 0.54–1.59, mean = 1.33). This suggests impaired synchronization between the more and less affected body sides during walking.
  - On a whole-body level (*n* = 4), patterns involving most body segments showed the largest deficits in the PD group (effect sizes: 1-1.04, mean = 1.02). This suggests that whole-body coordination in the ML direction, crucial for stability during walking, is affected by PD.

- *V direction* No significant differences were observed.

#### Fast walking speed

**Fig. 4:**
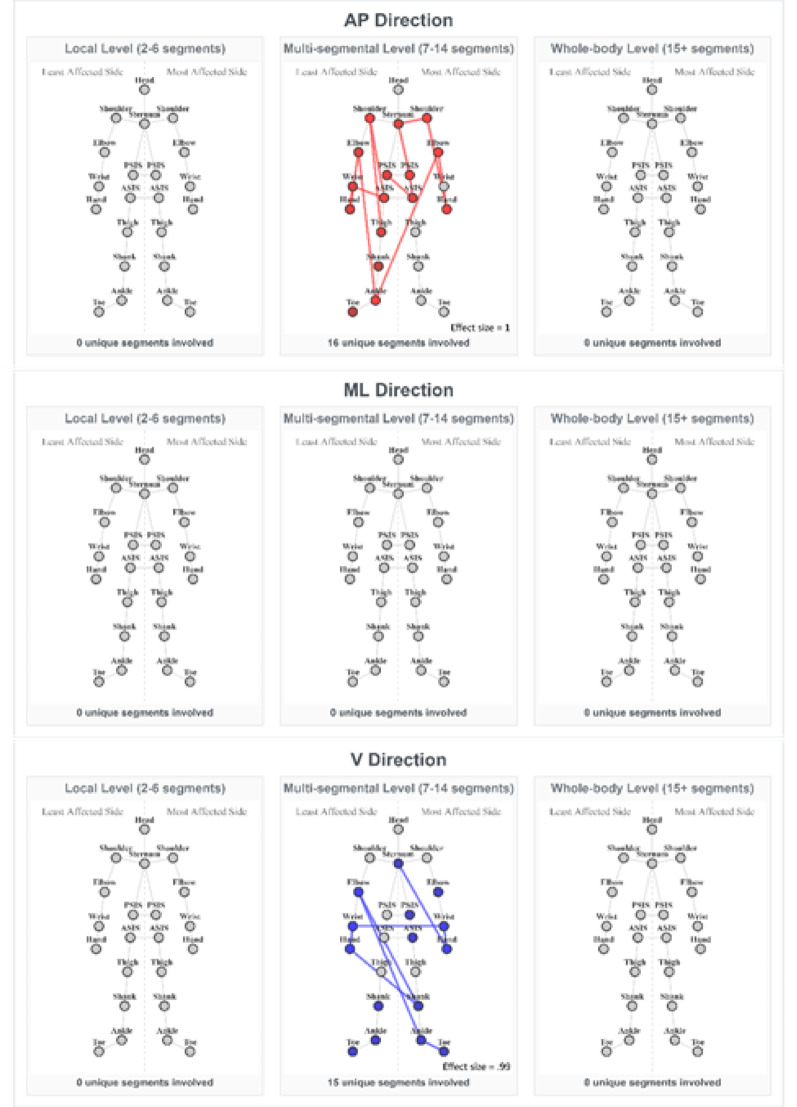
Significantly different coordination patterns between PD and controls at fast walking speed. The patterns with largest effect sizes are shown. Connected nodes indicate the strongest coordination deficit in pwPD; coloured but unconnected nodes appear in other significant patterns. Red indicates weaker, and blue indicates stronger inter-segmental coordination in the PD group than in the control group. The colour intensity reflects the frequency each node appeared in all significant patterns for the given speed, direction, and segmental level (more vivid - higher frequency). ASIS – anterior superior iliac spine; PSIS – posterior superior iliac spine

Fewer and distinct significant differences between the PD group and controls were observed compared to preferred speed, and they were observed exclusively on a multi-segmental level:

- *AP direction* Two patterns on a multi-segmental level were found to be significantly weaker in the PD group (effect sizes: 0.51-1, mean = 0.76) (Fig. 4, AP direction, subplot b). The patterns involved core segments, both upper limbs and the less affected lower limb, suggesting an impaired synchronization between the core and limb swing during walking.
- *ML direction* No significant differences.
- *V Direction* Two patterns on a multi-segmental level were found to be significantly different (Fig. 4, V direction, subplot b). The patterns involved sternum, most affected side of the pelvis and both upper and lower limbs. Interestingly, these values were *higher* in the PD group (effect sizes: 0.96-99, mean = 0.98). This suggests compensatory activation of muscles and potentially also increased rigidity within complex networks mainly including upper and lower limbs, as well as pelvic segments.

#### Slow walking speed

At slow walking speed, minimal significant differences in inter-segmental coordination were observed between the groups:

- *AP and V direction* No significant differences were found.
- *ML direction* One pattern was found to be different in the slow walking speed (Supplementary Fig. 2, ML direction, subplot a), involving the less affected shoulder and thigh, with an effect size of 1.17.

## Discussion

This study describes the inter-segmental coordination of the whole body during walking in PwPD and age-matched controls, and how this coordination changes at different walking speeds. The coordination between the segments was evaluated by the means of kinectomes, containing pairwise acceleration interactions between different body segments. Analysis methods were based on the network theory to evaluate the inter-segmental coordination differences between PwPD and controls [14].

The high correlations in the average kinectomes (Table 2) suggest that the fundamental inter-segmental coordination structure remains intact in PD. This result reflects our clinical experience: PwPD are able to propel themselves forward during their entire mobile phase of the disease, while maintaining the swing of contralateral limbs. As shown with our collected data [11] (Supplementary Fig. 1), as well as previous research, PwPD are also able to modulate their walking speed [40], although to a lesser extent than healthy older adults [7].

However, movement variability during gait, as seen in lower correlations in the standard deviation kinectomes (Table 2), as well as their overall higher standard deviation in the PD group (data not shown in the manuscript), was increased especially during the preferred walking speed. Increased variability in gait of PwPD has been previously shown by multiple studies, however they mostly investigated discrete spatial and temporal gait parameters [41] like swing time [40, 42], stride time [40, 43], stride length [44], and stride-to-stride variability [45]. Previous studies have also shown impairments in inter-limb coordination, however focusing on specific joint pairs [46, 47], or limb coordination [19, 48]. The results showed reduced ipsi- and contralateral inter-limb coordination in PwPD [46], and increased variability of arm swing phase relation [47]. However, the kinectome allows us to expand upon these findings by examining complex inter-segmental coordination (and the variability thereof) by analysing simultaneous kinematic relationships across all body segments and all three movement directions. This approach helps to dive deeper into pathological mechanisms during gait, but also to evaluate potential adaptability and compensatory mechanisms, providing insights for allied therapeutic health interventions [49, 50].

The analysis of strongest inter-segmental coordination patterns revealed the predominance of coordination deficits at preferred walking speed, suggesting that PwPD experience impaired motor control during their most comfortable walking speed, which is typically the most often used and most efficient for healthy people [51, 52]. Our results therefore suggest that in PwPD, the motor coordination system is more challenged during preferred than fast and slow walking speed. This consistent and multi-level (starting from pairs of two body segments and ending at patterns involving all body segments) deficit of inter-segmental coordination strength indicates fundamental impairments in integrating movements, which are crucial for dynamic stability [43] and overall gait execution. These coordination deficits likely reflect the underlying pathophysiology of PD where the degeneration of dopaminergic neurons affects the whole basal ganglia network [53], which is responsible for selecting optimal motor strategies [54]. In addition, the reduced coordination between the segments in PwPD, compared to controls, could make gait less efficient and require increased effort, contributing to further symptoms well known to occur in PD: fatigue [55] and reduced walking endurance [56].

Furthermore, the motor coordination deficits were most pronounced in the AP and ML directions. Deficits in the axial segments, especially in the AP direction, suggest issues with core postural control during ambulation, which is a known difficulty in PwPD [57]. Our data show that, in AP direction, the hip segments are centrally involved in significantly weaker patterns PwPD, suggesting that especially interventions targeting pelvic coordination (and its coordinated “interaction” with distant body parts) could help these people to keep their gait stable during forward movement. Furthermore, the decreased limb coordination in ML direction that we observed in our PD group, point to the presence of lateral stability impairments during gait in this disease. This is of particular relevance, as lateral stability deficits in PwPD have been linked to falls [58, 59].

Interestingly, the fast walking speed revealed a completely different picture of gait coordination pattern in PwPD. The patterns showed a shift from severely impaired gait coordination, pronounced in the AP and ML direction, as observed in the preferred walking condition, towards generally much “better” gait coordination during the fast walking condition. Previous studies have shown that, in PwPD, the variabilities of stride length and stride time are lower during fast walking than during preferred walking [60], bringing the gait pattern of PwPD during fast walking closer to that of healthy controls. We have observed similar results. During the fast walking condition, only two patterns in the AP direction and none in the ML direction were significantly weaker in the PD group than in the control group, as opposed to 146 significant patterns in the AP direction and 16 in the ML direction during preferred speed (Supplementary Table 2).

Why did we observe most differences of gait coordination between PD and controls in preferred walking speed? One explanation could be that walking at a speed above the “comfort zone” improves the arousal state [61] and with that resources to coordinate gait. Previously it was shown that a known phenomenon in PwPD called “paradoxical kinesis” occurs in situations when a person is highly influenced by external cues or higher arousal [61, 62] and is able to produce movements like healthy people. Another reason could be that higher walking speeds lead to a proportionally reduced energy consumption [52], potentially making brain reserves free for gait coordination. Moreover, PD is known to be a disease which affects automatic movements – PwPD lose previously acquired automatic skills and have difficulty restoring them [63]. Importantly, these automatic movements are associated with posterior putamen, a region in basal ganglia which is primarily affected by PD due to the loss of dopamine [64]. Preferred walking speed can be seen as automatic protocol for walking, and may therefore be particularly affected by PD. Fastwalking speed, as assessed in this study, could have served as the cue to move in a non-automated manner and demonstrate more control-like performance.

Why PwPD, based on the pattern analysis, showed an even higher inter-limb coordination than controls in the fast walking condition in the V direction, is not yet clear. We hypothesize that, to maintain stability and forward propulsion at higher walking speed, PwPD may stiffen multiple segments and put them to work together, sacrificing fluid, relaxed movements to increase stability and meet the more demanding walking speed requirements. Future studies should confirm our results and, if this is the case, investigate whether this high interlimb coordination in the V axis indeed reflects better coordination, or whether this adaptation comes with a loss of adaptability and thus an increased risk of negative consequences, such as falls.

The differences in gait coordination were least significant in the slow walking condition. We hypothesize that, at slow walking speed, the motor system has sufficient time to plan and execute movements, bringing the movement pattern closer to that observed in healthy adults. It is already known that, in order to compensate for reduced dynamic stability during walking, PwPD adopt strategies with shorter step length [65], which could have allowed them choosing a control-like inter-segmental integration on a whole-body level.

There are a few considerations worth mentioning regarding the pattern analysis. We have chosen to compare the maximum-weighted patterns between the groups as baseline, however future work examining weaker patterns could reveal compensatory mechanisms, fatigue, less optimal motor strategies, or subtle early stage changes during the disease progression. Furthermore, a sub-analysis of the patterns themselves could identify where do the strongest group patterns rank on an individual list, and if they are significantly stronger than the other possible patterns. In addition, future studies should inlcude measures of varability (e.g. by bootstrapping) to understand how generalizable the results from such high-level analysis are.

This study has several strengths and limitations. Among the strengths are the evaluation of inter-segmental coordination of the entire body, different walking speeds, and including bootstrapping to assess the stability of the kinectomes. The limitations include the evaluation of the PD group only after intake of medication. Different coordination patterns may be revealed in the off-medication state. Other limitations include high disease variability, the focus of relatively short bouts of straight walking, not including a second (cognitive) task, and a relatively high proportion of PwPD without lateralization. Furthermore, we did not control for cognitive influence (although the PD group showed well preserved cognitive function, with an average Montreal Cognitive Assessment score of 25 points) and the data was collected in a clinical setting, namely a movement analysis laboratory, with no obstacles and no disturbances, which certainly occur in usual daily lives.

## Conclusion

In conclusion, our results from a data-driven network analysis indicate that inter-segmental coordination of gait at preferred walking speed is severely affected in PwPD. We hypothesise that this alteration is due to deficits in automaticity and arousal networks. Furthermore, the distribution of the patterns that are significantly different from controls provide insights into the pathomechanistic aspects of body control during walking in PwPD, and potentially interesting treatment options. Interestingly, these coordination deficits were much less obvious during fast and slow walking, respectively. These findings support the implementation of different walking speeds in mobility training in PwPD. Our direction-specific analyses suggest that exercises which help in improving coordination in AP and ML directions, including contralateral limb swing and pelvic segmental coordination, have the most potential in addressing inter-limb coordination deficits in PD.

## Supplementary Information

Below is the link to the electronic supplementary material.

Supplementary Material 1.

## Data Availability

Part of the data analysed during the current study are available at [66]. The remaining data contains patient information and are available from the corresponding author on reasonable request. The data analysis code (https:/github.com/karsae/pykinectome) is available at [34]. It is platform independent, written in Python programming language. Requirements include Python version 3.11 or higher.
