# Supplementary material for: Inter-segmental coordination patterns in Parkinson’s disease are particularly disturbed during preferred walking speed: a data-driven network approach
Source: J Neuroeng Rehabil. 2025 Dec 12;23:39. doi: 10.1186/s12984-025-01835-1 (PMC12849571; doi:10.1186/s12984-025-01835-1)
Supplement: Supplementary file 1 — Supplementary Material 1. [file 12984_2025_1835_MOESM1_ESM.docx]

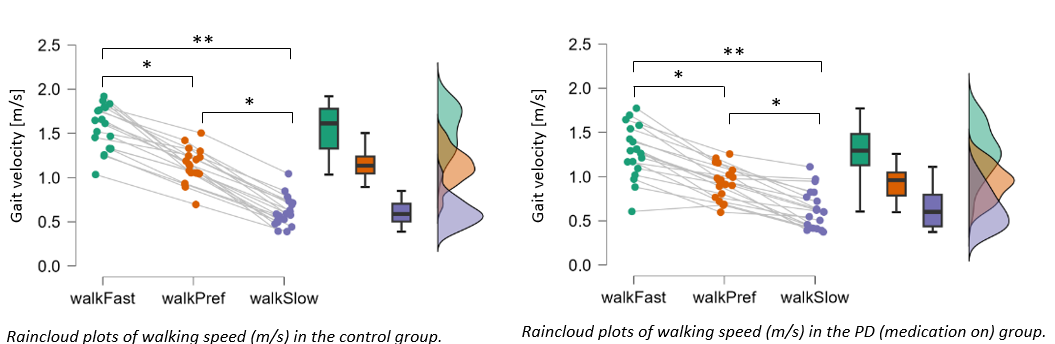
S-I. Differences in gait speed during walking trials*.*

**Supplementary Fig. 1** Fast (green), preferred (orange), and slow (purple) gait speed of controls (left) and pwPD (right)


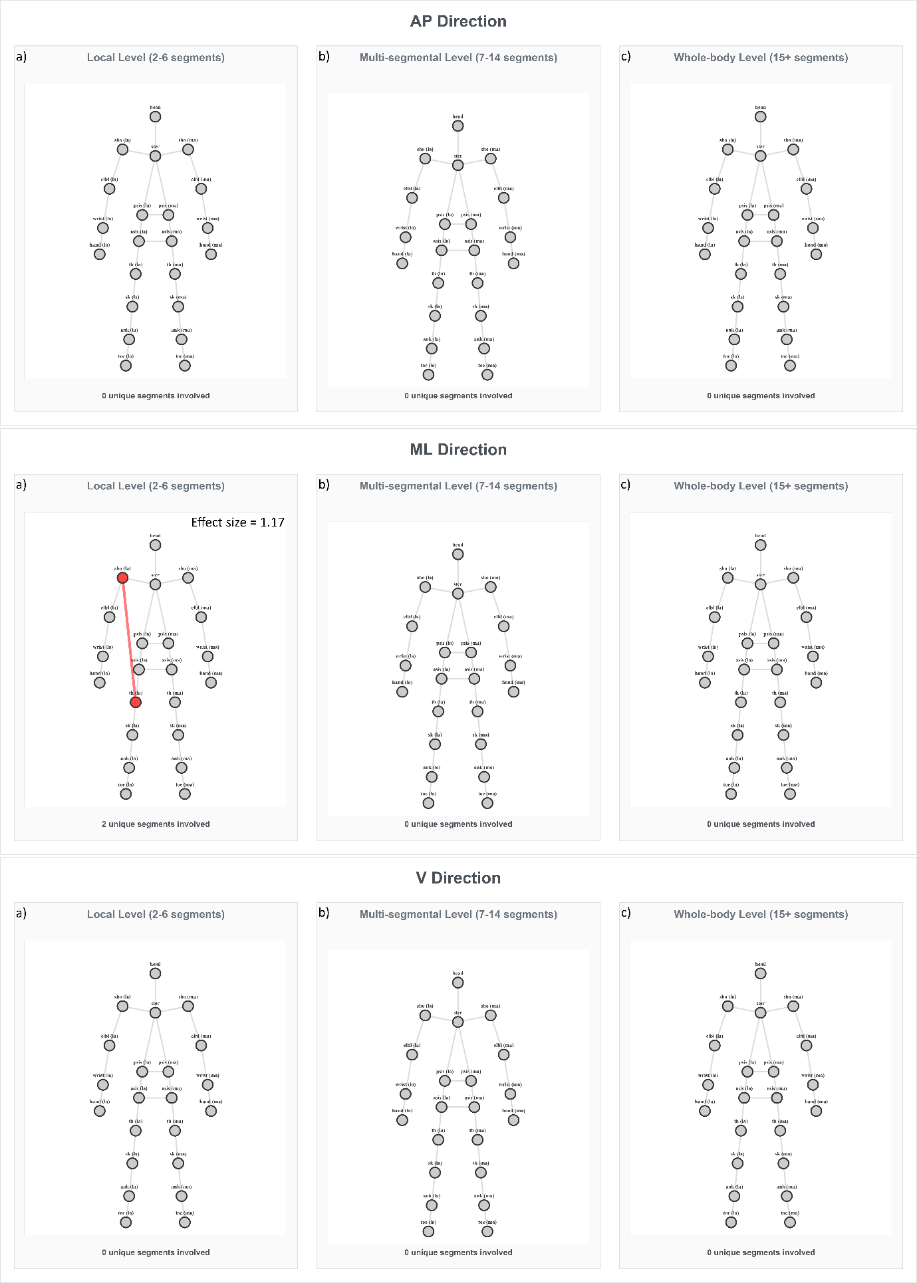
S-II. Inter-segmental coordination patterns in slow walking speed.

**Supplementary Fig. 2** Significantly different coordination pattern between PD and controls at slow walking speed. Connected nodes indicate the strongest coordination deficit in pwPD. Red indicates weaker inter-segmental coordination in the PD group than in the control group. ASIS – anterior superior iliac spine; PSIS – posterior superior iliac spine

S-III. Bootstrapping of the average kinectomes
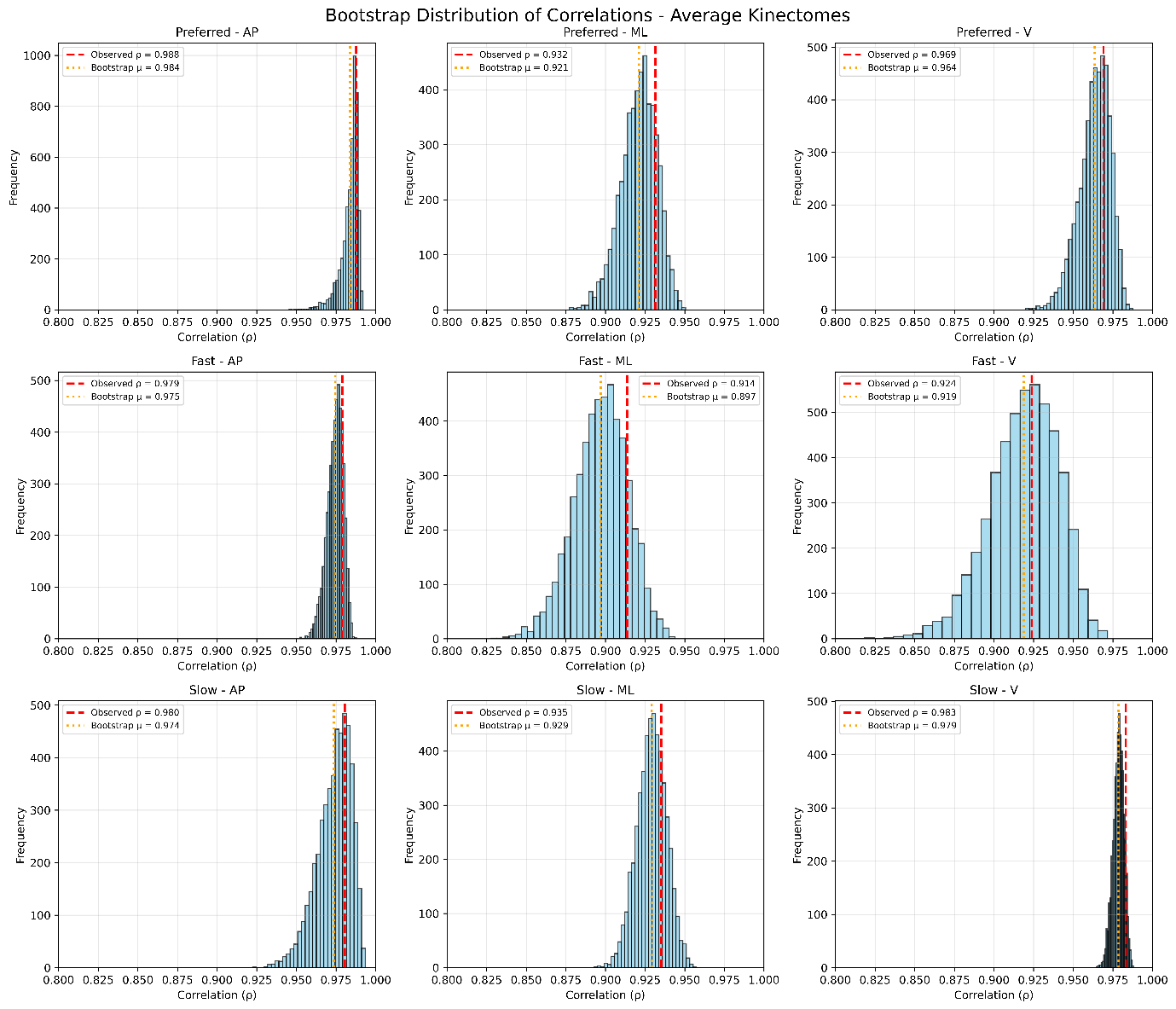


**Supplementary Fig. 3** Bootstrap distribution of correlations for the average kinectomes. The orange dashed line indicates the mean of the bootstrapped rho, and the red dashed line indicates the observed rho, both from permutation testing using Spearman’s correlation to evaluate the correlation between the group-specific average kinectomes

S-IV. Bootstrapping of the standard deviation kinectomes


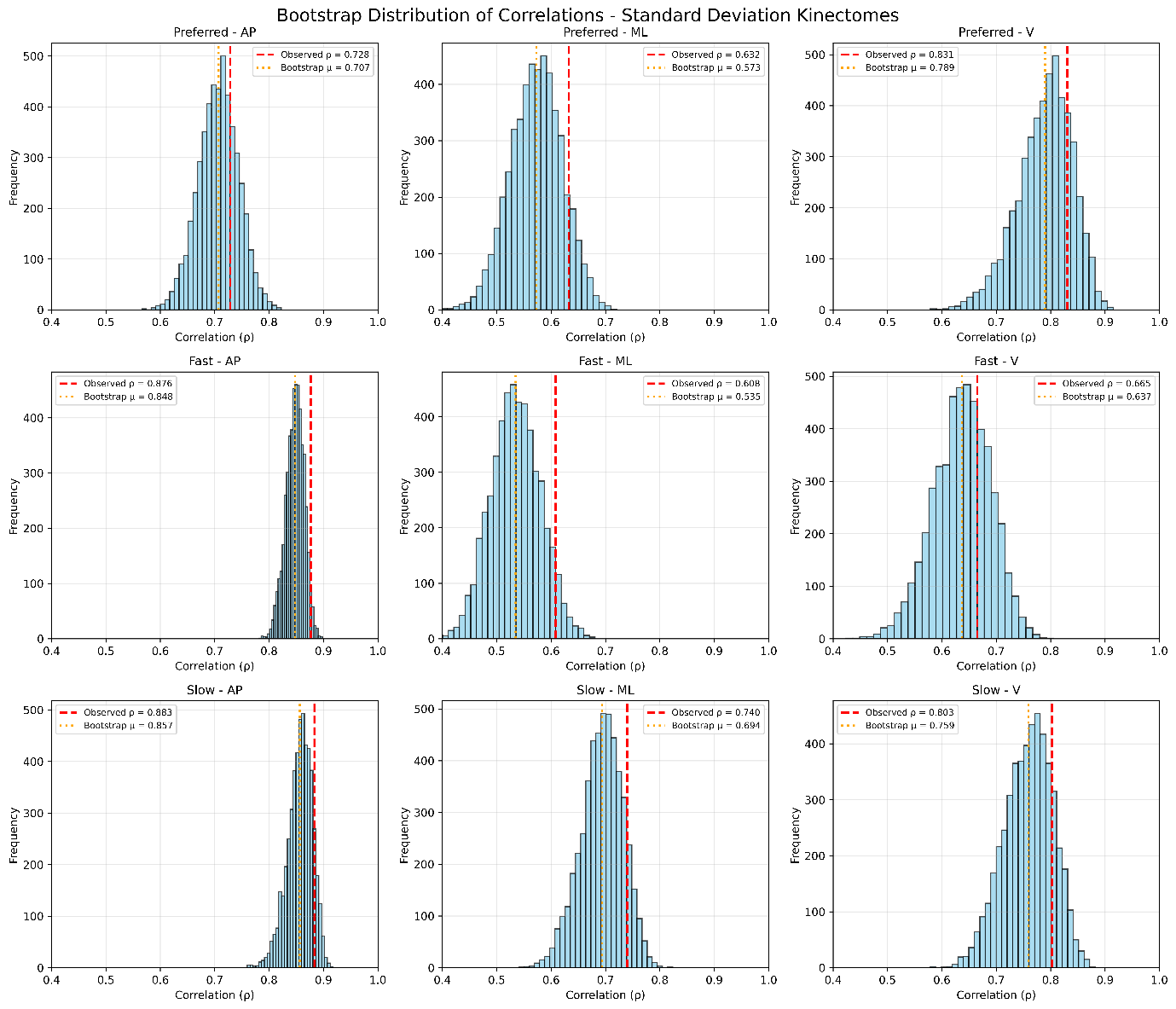


**Supplementary Fig. 4** Bootstrap distribution of correlations for the standard deviation kinectomes. The orange dashed line indicates the mean of the bootstrapped rho, and the red dashed line indicates the observed rho, both from permutation testing using Spearman’s correlation to evaluate the correlation between the group-specific standard deviation kinectomes

S-V. Strongest patterns found in the PD group, and their comparison with the same patterns in the control group

Supplementary Table 1. Strongest patterns found in the PD group

| Pattern | Pattern length | PD (mean ± SD) | Controls (mean ± SD) | p-value | Effect size |
| --- | --- | --- | --- | --- | --- |
| **Fast walking speed** | | | | | |
| AP direction | | | | | |
| ankle_mas-toe_mas-shoulder_las-head-shoulder_mas | 5 | 6.0 ± 0.4 | 5.5 ± 0.5 | <.001 | 1.1 |
| ML direction – no significant differences | | | | | |
| V direction | | | | | |
| thigh_mas-shank_mas-wrist_las-hand_las-psis_las-elbow_mas-wrist_mas | 7 | 8.2 ± 1.1 | 7.2 ± 0.9 | .02 | 1 |
| shoulder_mas-ankle_las-toe_las-shank_las-hand_mas-wrist_mas-asis_las-hand_las-shoulder_las-ankle_mas-shank_mas-wrist_las-elbow_las | 13 | 11.0 ± 1.9 | 9.2 ± 1.6 | .04 | -0.52 |
| shoulder_mas-ankle_las-toe_las-shank_las-hand_mas-wrist_mas-asis_las-hand_las-shoulder_las-ankle_mas-shank_mas-wrist_las-elbow_las-psis_mas | 14 | 12.5 ± 2.0 | 10.7 ± 1.8 | .04 | 0.95 |
| Preferred walking speed | | | | | |
| AP direction | | | | | |
| head-psis_mas | 2 | 1.3 ± 0.3 | 1.6 ± 0.3 | .04 | 0.56 |
| sternum-psis_las | 2 | 1.7 ± 0.1 | 1.8 ± 0.1 | <.001 | 0.75 |
| wrist_las-hand_las | 2 | 1.8 ± 0.1 | 1.9 ± 0.1 | .04 | 0.53 |
| asis_las-psis_las | 2 | 1.8 ± 0.1 | 1.9 ± 0.1 | <.001 | 0.65 |
| psis_mas-asis_las | 2 | 1.6 ± 0.2 | 1.8 ± 0.1 | .02 | 0.6 |
| head-psis_mas-psis_las | 3 | 3.0 ± 0.3 | 3.4 ± 0.3 | .02 | 0.58 |
| sternum-psis_las-psis_mas | 3 | 3.4 ± 0.3 | 3.7 ± 0.2 | <.001 | 0.69 |
| asis_las-psis_las-asis_mas | 3 | 3.4 ± 0.3 | 3.7 ± 0.2 | <.001 | 0.66 |
| psis_mas-asis_las-psis_las | 3 | 3.4 ± 0.3 | 3.7 ± 0.1 | <.001 | 0.64 |
| sternum-psis_las-psis_mas-asis_las | 4 | 5.0 ± 0.4 | 5.5 ± 0.3 | <.001 | 0.68 |
| hand_las-asis_las-psis_las-sternum | 4 | 4.0 ± 0.3 | 4.4 ± 0.3 | .04 | 1.33 |
| asis_las-psis_las-asis_mas-psis_mas | 4 | 5.2 ± 0.3 | 5.6 ± 0.2 | <.001 | 0.69 |
| head-psis_mas-asis_mas-psis_las-elbow_las | 5 | 5.7 ± 0.5 | 6.2 ± 0.5 | .02 | 1.0 |
| sternum-psis_las-asis_mas-asis_las-hand_las | 5 | 5.4 ± 0.5 | 6.0 ± 0.5 | <.001 | 0.62 |
| asis_las-psis_las-asis_mas-sternum-psis_mas | 5 | 6.7 ± 0.5 | 7.3 ± 0.3 | <.001 | 0.73 |
| psis_mas-asis_las-psis_las-sternum-shoulder_las | 5 | 6.6 ± 0.5 | 7.2 ± 0.3 | <.001 | 1.43 |
| head-psis_mas-asis_mas-psis_las-sternum-shoulder_mas | 6 | 8.1 ± 0.6 | 8.8 ± 0.5 | <.001 | 0.66 |
| sternum-psis_las-asis_mas-psis_mas-asis_las-hand_las | 6 | 7.3 ± 0.6 | 7.9 ± 0.5 | .02 | 1.08 |
| asis_las-psis_las-asis_mas-sternum-psis_mas-elbow_las | 6 | 7.7 ± 0.6 | 8.4 ± 0.3 | <.001 | 1.45 |
| psis_mas-asis_las-psis_las-sternum-asis_mas-hand_mas | 6 | 7.6 ± 0.7 | 8.3 ± 0.4 | <.001 | 1.21 |
| ankle_las-toe_las-shoulder_mas-asis_las-psis_las-psis_mas | 6 | 7.2 ± 0.6 | 7.9 ± 0.4 | <.001 | 0.67 |
| head-psis_mas-asis_mas-sternum-psis_las-asis_las-shoulder_mas | 7 | 9.6 ± 0.8 | 10.6 ± 0.6 | <.001 | 1.4 |
| sternum-psis_las-asis_mas-asis_las-hand_las-wrist_las-elbow_las | 7 | 8.9 ± 0.7 | 9.7 ± 0.6 | <.001 | 1.22 |
| shoulder_mas-ankle_las-toe_las-shank_las-elbow_mas-head-psis_mas | 7 | 7.6 ± 0.8 | 8.3 ± 0.8 | .02 | 0.57 |
| hand_las-asis_las-psis_las-psis_mas-sternum-shoulder_las-wrist_las | 7 | 8.3 ± 0.6 | 9.0 ± 0.6 | .04 | 1.17 |
| hand_mas-wrist_las-hand_las-elbow_las-psis_mas-asis_las-psis_las | 7 | 8.1 ± 0.7 | 8.5 ± 0.3 | .04 | 0.54 |
| asis_las-psis_las-asis_mas-sternum-psis_mas-elbow_las-shoulder_las | 7 | 9.1 ± 0.7 | 9.8 ± 0.4 | .02 | 1.21 |
| asis_mas-elbow_mas-wrist_mas-hand_mas-shank_las-sternum-psis_las | 7 | 8.7 ± 0.8 | 9.5 ± 0.8 | .04 | 0.56 |
| psis_las-asis_las-hand_las-wrist_las-thigh_las-shank_las-elbow_mas | 7 | 7.8 ± 0.5 | 8.3 ± 0.4 | .02 | 1.1 |
| psis_mas-asis_las-psis_las-sternum-shoulder_mas-thigh_las-shank_las | 7 | 9.7 ± 0.7 | 10.6 ± 0.4 | <.001 | 1.55 |
| ankle_las-toe_las-shoulder_mas-head-psis_mas-sternum-shoulder_las | 7 | 8.6 ± 0.6 | 9.2 ± 0.6 | <.001 | 0.65 |
| ankle_mas-toe_mas-shoulder_las-psis_mas-psis_las-sternum-head | 7 | 8.7 ± 0.6 | 9.3 ± 0.4 | <.001 | 1.16 |
| toe_las-ankle_las-shoulder_mas-sternum-psis_mas-elbow_mas-wrist_mas | 7 | 8.8 ± 0.5 | 9.3 ± 0.5 | .04 | 1.0 |
| shoulder_mas-ankle_las-toe_las-shank_las-elbow_mas-head-psis_mas-asis_mas | 8 | 9.4 ± 0.8 | 10.2 ± 0.8 | .02 | 0.58 |
| hand_las-asis_las-psis_las-psis_mas-sternum-shoulder_las-wrist_las-elbow_las | 8 | 10.0 ± 0.8 | 10.8 ± 0.7 | .04 | 1.06 |
| asis_mas-elbow_mas-wrist_mas-hand_mas-shank_las-thigh_las-psis_mas-asis_las | 8 | 10.6 ± 0.9 | 11.5 ± 0.9 | .02 | 0.58 |
| psis_las-asis_las-hand_las-wrist_las-thigh_las-shank_las-elbow_mas-hand_mas | 8 | 9.4 ± 0.7 | 10.0 ± 0.5 | .04 | 0.98 |
| psis_mas-asis_las-psis_las-sternum-shoulder_mas-thigh_las-shank_las-hand_mas | 8 | 11.0 ± 1.0 | 12.1 ± 0.9 | <.001 | 0.62 |
| shank_las-wrist_mas-hand_mas-thigh_mas-asis_mas-psis_mas-shoulder_las-psis_las | 8 | 9.6 ± 0.8 | 10.4 ± 0.6 | .02 | 1.12 |
| ankle_las-toe_las-shoulder_mas-head-psis_las-hand_las-shank_las-elbow_mas | 8 | 7.7 ± 0.5 | 8.2 ± 0.5 | .02 | 1.0 |
| ankle_mas-toe_mas-shoulder_las-psis_mas-head-sternum-asis_las-shoulder_mas | 8 | 9.6 ± 0.9 | 10.6 ± 0.8 | <.001 | 0.65 |
| head-psis_mas-asis_mas-psis_las-sternum-shoulder_mas-shoulder_las-ankle_mas-toe_mas | 9 | 11.9 ± 0.8 | 12.7 ± 0.7 | .04 | 0.54 |
| shoulder_mas-ankle_las-toe_las-shank_las-elbow_mas-head-psis_mas-asis_mas-psis_las | 9 | 11.0 ± 0.8 | 12.0 ± 0.9 | <.001 | 0.62 |
| hand_las-asis_las-psis_las-sternum-psis_mas-asis_mas-hand_mas-wrist_mas-elbow_mas | 9 | 12.0 ± 0.8 | 12.8 ± 0.6 | .02 | 1.12 |
| asis_mas-elbow_mas-wrist_mas-hand_mas-shank_las-thigh_las-shoulder_mas-psis_las-asis_las | 9 | 12.2 ± 1.0 | 13.3 ± 1.0 | .02 | 0.58 |
| psis_las-asis_las-hand_las-wrist_las-thigh_las-elbow_mas-hand_mas-ankle_las-toe_las | 9 | 10.7 ± 0.8 | 11.5 ± 0.6 | .02 | 1.12 |
| shank_las-wrist_mas-hand_mas-thigh_mas-asis_mas-psis_mas-shoulder_las-sternum-asis_las | 9 | 11.5 ± 0.8 | 12.3 ± 0.6 | <.001 | 0.61 |
| ankle_las-toe_las-shoulder_mas-head-psis_las-hand_las-shank_las-elbow_mas-wrist_mas | 9 | 9.4 ± 0.6 | 10.0 ± 0.6 | <.001 | 1.0 |
| toe_las-ankle_las-shoulder_mas-asis_las-psis_las-hand_las-wrist_las-shank_las-elbow_mas | 9 | 9.9 ± 0.6 | 10.7 ± 0.5 | <.001 | 1.44 |
| toe_mas-ankle_mas-shoulder_las-psis_mas-asis_las-psis_las-sternum-shoulder_mas-head | 9 | 12.2 ± 0.8 | 13.0 ± 0.6 | .04 | 0.55 |
| head-psis_mas-asis_mas-psis_las-sternum-shoulder_mas-shoulder_las-ankle_mas-toe_mas-hand_las | 10 | 13.2 ± 1.0 | 14.1 ± 0.9 | .04 | 0.54 |
| shank_las-wrist_mas-hand_mas-thigh_mas-asis_mas-psis_mas-shoulder_las-psis_las-asis_las-elbow_mas | 10 | 12.7 ± 1.0 | 13.7 ± 0.8 | .02 | 1.1 |
| ankle_las-toe_las-shoulder_mas-head-psis_las-hand_las-shank_las-elbow_mas-wrist_mas-hand_mas | 10 | 11.2 ± 0.6 | 12.0 ± 0.6 | <.001 | 1.33 |
| ankle_mas-toe_mas-shoulder_las-psis_mas-asis_las-shoulder_mas-head-sternum-psis_las-hand_las | 10 | 12.2 ± 1.0 | 13.1 ± 0.9 | .02 | 0.94 |
| toe_las-ankle_las-shoulder_mas-asis_las-psis_las-hand_las-wrist_las-shank_las-elbow_mas-wrist_mas | 10 | 11.6 ± 0.7 | 12.5 ± 0.5 | <.001 | 1.46 |
| hand_las-asis_las-psis_las-asis_mas-hand_mas-wrist_mas-elbow_mas-ankle_las-shank_las-thigh_las-shoulder_las | 11 | 13.6 ± 0.9 | 14.7 ± 0.7 | <.001 | 0.64 |
| psis_mas-asis_las-hand_las-wrist_las-thigh_las-shoulder_las-shoulder_mas-ankle_las-toe_las-elbow_mas-hand_mas | 11 | 12.0 ± 0.8 | 12.9 ± 0.6 | <.001 | 1.26 |
| ankle_las-toe_las-shoulder_mas-head-psis_mas-asis_las-psis_las-hand_las-shank_las-elbow_mas-wrist_mas | 11 | 12.7 ± 0.8 | 13.7 ± 0.6 | <.001 | 1.4 |
| ankle_mas-toe_mas-shoulder_las-head-sternum-psis_mas-asis_las-shoulder_mas-psis_las-hand_las-shank_mas | 11 | 13.6 ± 1.3 | 14.8 ± 1.0 | .04 | 1.03 |
| toe_las-ankle_las-shoulder_mas-head-psis_mas-asis_las-psis_las-hand_las-sternum-shoulder_las-asis_mas | 11 | 12.9 ± 1.0 | 14.1 ± 1.0 | <.001 | 1.2 |
| sternum-psis_las-asis_las-hand_las-wrist_las-elbow_las-head-shoulder_mas-ankle_las-toe_las-hand_mas-ankle_mas | 12 | 14.5 ± 0.8 | 15.3 ± 0.7 | <.001 | 0.65 |
| elbow_mas-shank_las-hand_mas-ankle_mas-shoulder_las-head-psis_mas-asis_mas-psis_las-sternum-shoulder_mas-ankle_las | 12 | 14.3 ± 1.3 | 15.4 ± 1.1 | .02 | 0.57 |
| hand_las-asis_las-psis_las-asis_mas-hand_mas-wrist_mas-elbow_mas-ankle_las-shoulder_mas-head-shoulder_las-wrist_las | 12 | 14.4 ± 1.1 | 15.4 ± 1.0 | .04 | 0.95 |
| psis_mas-asis_las-psis_las-sternum-shoulder_mas-ankle_las-toe_las-shoulder_las-head-asis_mas-hand_mas-wrist_mas | 12 | 15.2 ± 0.7 | 16.4 ± 0.7 | <.001 | 1.71 |
| shank_las-wrist_mas-hand_mas-ankle_mas-shoulder_las-psis_las-asis_las-thigh_las-shoulder_mas-ankle_las-toe_las-head | 12 | 13.2 ± 1.0 | 14.2 ± 0.8 | .04 | 0.55 |
| ankle_las-toe_las-shoulder_mas-head-psis_mas-asis_las-psis_las-hand_las-shank_las-elbow_mas-wrist_mas-hand_mas | 12 | 14.6 ± 0.8 | 15.6 ± 0.7 | <.001 | 1.32 |
| head-psis_mas-asis_mas-psis_las-sternum-shoulder_mas-shoulder_las-ankle_mas-shank_mas-wrist_las-hand_las-elbow_las-shank_las | 13 | 17.0 ± 1.1 | 18.0 ± 0.9 | .04 | 0.55 |
| sternum-psis_las-asis_las-hand_las-wrist_las-elbow_las-head-shoulder_mas-ankle_las-toe_las-hand_mas-wrist_mas-shank_las | 13 | 17.1 ± 1.4 | 18.3 ± 1.2 | .04 | 0.55 |
| wrist_mas-hand_mas-shank_las-hand_las-wrist_las-thigh_las-shoulder_mas-ankle_las-toe_las-elbow_las-shank_mas-thigh_mas-asis_mas | 13 | 14.6 ± 0.8 | 15.3 ± 0.6 | .02 | 0.58 |
| hand_las-asis_las-psis_las-sternum-psis_mas-asis_mas-hand_mas-wrist_mas-elbow_mas-ankle_las-shoulder_mas-head-shoulder_las | 13 | 16.9 ± 1.1 | 18.2 ± 0.9 | <.001 | 1.28 |
| asis_las-psis_las-asis_mas-hand_mas-ankle_las-toe_las-shoulder_mas-head-psis_mas-sternum-shoulder_las-ankle_mas-toe_mas | 13 | 16.5 ± 1.1 | 17.8 ± 1.0 | <.001 | 0.62 |
| asis_mas-elbow_mas-hand_mas-wrist_mas-shank_las-hand_las-wrist_las-elbow_las-shank_mas-thigh_mas-head-asis_las-shoulder_mas | 13 | 16.7 ± 1.4 | 18.2 ± 1.2 | .02 | 0.6 |
| psis_mas-asis_las-psis_las-sternum-shoulder_mas-ankle_las-toe_las-shoulder_las-head-asis_mas-hand_mas-wrist_mas-shank_las | 13 | 16.5 ± 1.0 | 18.0 ± 1.1 | <.001 | 1.43 |
| ankle_las-toe_las-shoulder_mas-head-psis_mas-asis_las-psis_las-hand_las-shank_las-elbow_mas-hand_mas-wrist_mas-wrist_las | 13 | 14.7 ± 0.9 | 15.6 ± 0.7 | .02 | 1.11 |
| ankle_mas-toe_mas-shoulder_las-head-sternum-psis_mas-thigh_mas-shank_mas-wrist_las-elbow_las-psis_las-asis_las-shoulder_mas | 13 | 17.3 ± 1.2 | 18.4 ± 1.0 | .04 | 0.99 |
| toe_mas-ankle_mas-shoulder_las-head-asis_las-psis_mas-sternum-psis_las-hand_las-shank_mas-wrist_las-elbow_las-toe_las | 13 | 15.8 ± 1.4 | 17.0 ± 1.2 | .04 | 0.92 |
| head-psis_mas-asis_mas-psis_las-asis_las-shoulder_mas-sternum-shoulder_las-ankle_mas-shank_mas-wrist_las-elbow_las-shank_las-wrist_mas | 14 | 18.2 ± 1.4 | 19.7 ± 1.2 | <.001 | 0.61 |
| hand_las-asis_las-psis_las-sternum-psis_mas-asis_mas-hand_mas-wrist_mas-thigh_las-shank_las-head-shoulder_las-wrist_las-elbow_las | 14 | 18.5 ± 1.3 | 19.8 ± 1.0 | <.001 | 1.11 |
| asis_mas-elbow_mas-hand_mas-wrist_mas-shank_las-hand_las-wrist_las-elbow_las-shank_mas-thigh_mas-head-asis_las-shoulder_mas-ankle_las | 14 | 17.4 ± 1.4 | 19.0 ± 1.2 | .02 | 0.61 |
| toe_las-ankle_las-shoulder_mas-head-psis_mas-asis_las-psis_las-hand_las-sternum-shoulder_las-asis_mas-hand_mas-shank_las-elbow_mas | 14 | 16.4 ± 1.7 | 18.1 ± 1.5 | .02 | 1.06 |
| toe_mas-ankle_mas-shoulder_las-head-asis_las-psis_mas-sternum-psis_las-hand_las-shank_mas-wrist_las-elbow_las-toe_las-ankle_las | 14 | 17.6 ± 1.4 | 18.9 ± 1.2 | .04 | 0.99 |
| elbow_mas-shank_las-hand_mas-wrist_mas-thigh_las-shoulder_mas-ankle_las-toe_las-head-psis_mas-asis_mas-sternum-shoulder_las-hand_las-wrist_las | 15 | 19.1 ± 2.2 | 20.9 ± 2.2 | .02 | 0.58 |
| asis_las-psis_las-asis_mas-hand_mas-wrist_mas-shank_las-hand_las-wrist_las-elbow_las-shank_mas-thigh_mas-psis_mas-sternum-shoulder_mas-head | 15 | 20.7 ± 1.2 | 21.9 ± 1.0 | .02 | 1.08 |
| asis_mas-elbow_mas-hand_mas-wrist_mas-thigh_las-shoulder_mas-ankle_las-toe_las-shank_las-hand_las-wrist_las-elbow_las-shank_mas-thigh_mas-head | 15 | 19.0 ± 1.4 | 20.3 ± 1.2 | .04 | 0.54 |
| shank_mas-hand_mas-wrist_mas-shank_las-elbow_mas-ankle_las-shoulder_mas-asis_mas-psis_mas-sternum-psis_las-head-shoulder_las-ankle_mas-toe_mas | 15 | 18.8 ± 1.7 | 20.4 ± 1.5 | .04 | 0.56 |
| toe_mas-ankle_mas-shoulder_las-head-asis_las-psis_mas-sternum-psis_las-hand_las-shank_mas-wrist_las-elbow_las-toe_las-ankle_las-thigh_las | 15 | 18.5 ± 1.5 | 19.9 ± 1.2 | .04 | 1.02 |
| elbow_mas-shank_las-hand_mas-wrist_mas-thigh_las-shoulder_mas-ankle_las-psis_las-sternum-psis_mas-asis_mas-asis_las-hand_las-ankle_mas-shoulder_las-head | 16 | 19.1 ± 2.8 | 21.1 ± 2.6 | .04 | 0.53 |
| wrist_mas-hand_mas-shank_las-elbow_mas-shank_mas-wrist_las-hand_las-ankle_las-toe_las-shoulder_las-asis_mas-sternum-psis_mas-psis_las-asis_las-shoulder_mas | 16 | 20.6 ± 1.4 | 22.2 ± 1.1 | <.001 | 1.26 |
| hand_mas-wrist_las-elbow_mas-shank_las-thigh_las-shoulder_mas-ankle_las-toe_las-shoulder_las-head-sternum-psis_mas-asis_mas-wrist_mas-thigh_mas-shank_mas | 16 | 17.7 ± 1.3 | 18.4 ± 1.0 | .04 | 0.55 |
| asis_las-psis_las-asis_mas-hand_mas-wrist_mas-shank_las-hand_las-wrist_las-elbow_las-shank_mas-thigh_mas-psis_mas-sternum-shoulder_mas-head-shoulder_las | 16 | 22.3 ± 1.3 | 23.5 ± 1.1 | .04 | 0.99 |
| asis_mas-elbow_mas-hand_mas-wrist_mas-thigh_las-shoulder_mas-ankle_las-toe_las-shank_las-hand_las-wrist_las-elbow_las-shank_mas-thigh_mas-head-asis_las | 16 | 20.2 ± 1.4 | 21.8 ± 1.3 | .02 | 0.59 |
| shank_mas-hand_mas-wrist_mas-shank_las-elbow_mas-ankle_las-shoulder_mas-asis_mas-psis_mas-sternum-psis_las-head-shoulder_las-ankle_mas-toe_mas-thigh_las | 16 | 19.0 ± 1.7 | 20.5 ± 1.5 | .04 | 0.53 |
| sternum-psis_las-asis_las-hand_las-wrist_las-elbow_las-shank_mas-hand_mas-wrist_mas-shank_las-head-shoulder_mas-ankle_las-elbow_mas-psis_mas-asis_mas-shoulder_las | 17 | 21.4 ± 1.7 | 23.0 ± 1.4 | .04 | 0.55 |
| elbow_mas-shank_las-hand_mas-wrist_mas-thigh_las-shoulder_mas-ankle_las-psis_las-sternum-psis_mas-asis_mas-asis_las-hand_las-wrist_las-ankle_mas-shoulder_las-head | 17 | 21.0 ± 2.8 | 23.0 ± 2.7 | .04 | 0.54 |
| wrist_mas-hand_mas-shank_las-hand_las-ankle_las-toe_las-shoulder_las-asis_mas-sternum-psis_mas-psis_las-asis_las-shoulder_mas-head-toe_mas-ankle_mas-shank_mas | 17 | 21.2 ± 1.2 | 22.4 ± 1.1 | .02 | 1.04 |
| asis_mas-elbow_mas-hand_mas-wrist_mas-thigh_las-shoulder_mas-ankle_las-toe_las-shank_las-hand_las-wrist_las-elbow_las-shank_mas-thigh_mas-head-asis_las-psis_las | 17 | 22.0 ± 1.5 | 23.7 ± 1.3 | .02 | 0.6 |
| shank_mas-hand_mas-wrist_mas-shank_las-elbow_mas-ankle_las-shoulder_mas-asis_mas-psis_mas-sternum-psis_las-head-shoulder_las-ankle_mas-toe_mas-thigh_las-asis_las | 17 | 20.5 ± 1.8 | 22.1 ± 1.6 | .04 | 0.54 |
| asis_mas-elbow_mas-hand_mas-wrist_mas-thigh_las-shoulder_mas-ankle_las-toe_las-shank_las-hand_las-wrist_las-ankle_mas-shoulder_las-psis_mas-sternum-asis_las-psis_las-head | 18 | 22.8 ± 1.6 | 24.7 ± 1.4 | <.001 | 1.26 |
| hand_mas-wrist_las-hand_las-elbow_las-shank_las-elbow_mas-ankle_las-psis_las-sternum-psis_mas-asis_mas-wrist_mas-thigh_mas-shoulder_las-head-shoulder_mas-asis_las-thigh_las-toe_las | 19 | 22.1 ± 1.5 | 23.5 ± 1.2 | .02 | 0.56 |
| shank_mas-hand_mas-wrist_mas-shank_las-elbow_mas-psis_mas-sternum-psis_las-head-shoulder_mas-asis_mas-asis_las-hand_las-wrist_las-elbow_las-ankle_mas-toe_mas-shoulder_las-ankle_las | 19 | 23.7 ± 1.7 | 25.3 ± 1.5 | .04 | 0.99 |
| shank_mas-hand_mas-wrist_mas-shank_las-elbow_mas-psis_mas-sternum-psis_las-head-shoulder_mas-asis_mas-asis_las-hand_las-wrist_las-elbow_las-ankle_mas-toe_mas-shoulder_las-ankle_las-toe_las | 20 | 25.5 ± 1.7 | 27.2 ± 1.6 | .04 | 1.03 |
| ML direction | | | | | |
| elbow_las-shank_mas-wrist_las-hand_las-ankle_las | 5 | 3.1 ± 0.5 | 3.7 ± 0.5 | .02 | 1.2 |
| thigh_las-hand_mas-thigh_mas-elbow_las-shank_mas-wrist_las-hand_las-ankle_las-toe_las-head | 10 | 6.7 ± 0.9 | 7.7 ± 0.9 | .02 | 1.11 |
| thigh_las-hand_mas-thigh_mas-elbow_las-shank_mas-wrist_las-hand_las-ankle_las-toe_las-head-shoulder_mas-asis_las | 12 | 9.1 ± 1.0 | 10.3 ± 1.0 | <.001 | 1.2 |
| wrist_mas-hand_mas-shank_las-hand_las-wrist_las-thigh_las-shoulder_mas-ankle_las-toe_las-elbow_las-shank_mas-thigh_mas-asis_mas | 13 | 10.3 ± 0.9 | 11.3 ± 0.9 | <.001 | 1.11 |
| wrist_mas-hand_mas-shank_las-hand_las-ankle_las-toe_las-elbow_las-shank_mas-wrist_las-thigh_las-shoulder_mas-head-toe_mas-ankle_mas | 14 | 10.5 ± 0.9 | 11.6 ± 0.9 | .02 | 1.22 |
| asis_mas-elbow_mas-hand_mas-wrist_mas-thigh_las-shoulder_mas-ankle_las-toe_las-shank_las-hand_las-wrist_las-elbow_las-shank_mas-thigh_mas-head | 15 | 12.8 ± 0.9 | 13.9 ± 1.2 | .02 | 1.05 |
| asis_mas-elbow_mas-hand_mas-wrist_mas-thigh_las-shoulder_mas-ankle_las-toe_las-shank_las-hand_las-wrist_las-elbow_las-shank_mas-thigh_mas-head-asis_las | 16 | 13.7 ± 1.0 | 15.0 ± 1.2 | .02 | 1.18 |
| asis_mas-elbow_mas-hand_mas-wrist_mas-thigh_las-shoulder_mas-ankle_las-toe_las-shank_las-hand_las-wrist_las-elbow_las-shank_mas-thigh_mas-head-asis_las-psis_las | 17 | 14.6 ± 1.1 | 15.9 ± 1.5 | .04 | 1.0 |
| V direction – no significant differences | | | | | |
| **Slow walking speed** | | | | | |
| AP direction – no significant differences | | | | | |
| ML direction – no significant differences | | | | | |
| V direction – no significant differences | | | | | |

S-VI. Strongest patterns found in the control group, and their comparison with the same patterns in the PD group.

Supplementary Table 2. Strongest patterns found in the control group

| Pattern | Pattern length | PD group (mean ± SD) | Control group (mean ± SD) | p-value | Effect size |
| --- | --- | --- | --- | --- | --- |
| **Fast walking speed** | | | | | |
| AP direction | | | | | |
| shank_las-hand_mas-elbow_mas-ankle_las-toe_las-shoulder_mas-thigh_las-shoulder_las-asis_las-psis_mas | 10 | 12.1 ± 1.4 | 13.2 ± 1.8 | .04 | 0.51 |
| psis_las-asis_mas-psis_mas-sternum-shoulder_mas-hand_mas-elbow_mas-ankle_las-elbow_las-hand_las-wrist_las-asis_las-shoulder_las-thigh_las | 14 | 17.7 ± 1.1 | 18.8 ± 1.1 | .02 | 1.0 |
| ML direction – no significant differences | | | | | |
| V direction | | | | | |
| toe_mas-ankle_mas-elbow_las-shank_mas-hand_las-wrist_las-wrist_mas-hand_mas-sternum | 9 | 8.5 ± 1.1 | 7.6 ± 0.7 | .02 | 0.99 |
| elbow_mas-hand_mas-shank_las-wrist_mas-ankle_las-toe_las-elbow_las-hand_las-wrist_las-asis_mas-psis_mas | 11 | 10.8 ± 1.5 | 9.5 ± 1.2 | .04 | 0.96 |
| **Preferred walking speed** | | | | | |
| AP direction | | | | | |
| head-psis_las | 2 | 1.3 ± 0.3 | 1.6 ± 0.2 | .04 | 0.55 |
| sternum-psis_mas | 2 | 1.7 ± 0.2 | 1.8 ± 0.1 | <.001 | 0.71 |
| wrist_las-hand_las | 2 | 1.8 ± 0.1 | 1.9 ± 0.1 | .04 | 0.53 |
| asis_las-psis_mas | 2 | 1.6 ± 0.2 | 1.8 ± 0.1 | .02 | 0.6 |
| asis_mas-psis_las | 2 | 1.6 ± 0.2 | 1.8 ± 0.1 | <.001 | 0.62 |
| psis_mas-asis_mas | 2 | 1.8 ± 0.1 | 1.9 ± 0.1 | <.001 | 0.67 |
| head-psis_las-asis_las | 3 | 3.1 ± 0.3 | 3.5 ± 0.3 | <.001 | 0.68 |
| sternum-psis_mas-asis_mas | 3 | 3.5 ± 0.2 | 3.7 ± 0.1 | <.001 | 0.72 |
| asis_las-psis_mas-asis_mas | 3 | 3.4 ± 0.2 | 3.7 ± 0.1 | <.001 | 1.86 |
| psis_las-asis_mas-psis_mas | 3 | 3.4 ± 0.2 | 3.7 ± 0.2 | <.001 | 0.65 |
| head-psis_las-asis_las-psis_mas | 4 | 4.7 ± 0.5 | 5.2 ± 0.3 | <.001 | 1.19 |
| psis_las-asis_mas-head-sternum | 4 | 4.5 ± 0.6 | 5.0 ± 0.5 | .02 | 0.58 |
| psis_mas-asis_mas-sternum-psis_las | 4 | 5.1 ± 0.3 | 5.5 ± 0.2 | <.001 | 0.74 |
| head-psis_las-asis_las-psis_mas-asis_mas | 5 | 6.5 ± 0.5 | 7.1 ± 0.4 | <.001 | 1.32 |
| shoulder_las-thigh_las-shank_las-wrist_mas-hand_mas | 5 | 5.9 ± 0.6 | 6.5 ± 0.6 | .04 | 0.54 |
| asis_mas-psis_las-asis_las-sternum-shoulder_mas | 5 | 6.7 ± 0.5 | 7.3 ± 0.3 | <.001 | 0.74 |
| psis_mas-asis_mas-sternum-psis_las-asis_las | 5 | 6.9 ± 0.4 | 7.4 ± 0.3 | <.001 | 0.77 |
| toe_las-psis_las-head-psis_mas-asis_mas | 5 | 4.9 ± 0.6 | 5.5 ± 0.5 | <.001 | 0.65 |
| head-psis_las-asis_las-psis_mas-asis_mas-shoulder_las | 6 | 7.8 ± 0.8 | 8.7 ± 0.5 | <.001 | 0.69 |
| sternum-psis_mas-head-shoulder_las-asis_mas-psis_las | 6 | 7.5 ± 0.9 | 8.4 ± 0.6 | <.001 | 0.61 |
| shoulder_las-thigh_las-shank_las-wrist_mas-hand_mas-elbow_mas | 6 | 7.5 ± 0.8 | 8.2 ± 0.7 | .04 | 0.53 |
| hand_mas-wrist_mas-ankle_las-toe_las-shoulder_mas-thigh_las | 6 | 6.7 ± 0.9 | 7.4 ± 0.9 | .02 | 0.58 |
| psis_mas-asis_mas-sternum-psis_las-asis_las-shoulder_las | 6 | 8.2 ± 0.6 | 9.0 ± 0.3 | <.001 | 1.65 |
| thigh_las-shoulder_las-psis_mas-asis_mas-psis_las-sternum | 6 | 7.5 ± 0.7 | 8.3 ± 0.5 | <.001 | 1.3 |
| thigh_mas-shoulder_mas-asis_mas-psis_las-head-psis_mas | 6 | 6.7 ± 1.0 | 7.5 ± 0.9 | .02 | 0.56 |
| toe_las-psis_las-head-psis_mas-asis_mas-asis_las | 6 | 6.4 ± 0.7 | 7.3 ± 0.5 | <.001 | 0.69 |
| elbow_mas-hand_mas-wrist_mas-shank_las-thigh_las-shoulder_mas-asis_mas | 7 | 9.2 ± 1.0 | 10.1 ± 0.9 | .02 | 0.59 |
| hand_mas-wrist_mas-ankle_las-toe_las-shoulder_mas-shank_las-elbow_mas | 7 | 7.9 ± 1.5 | 8.8 ± 1.5 | .04 | 0.54 |
| asis_las-psis_mas-asis_mas-sternum-psis_las-shoulder_las-thigh_mas | 7 | 9.2 ± 0.9 | 10.3 ± 0.6 | <.001 | 0.71 |
| psis_mas-asis_mas-psis_las-asis_las-sternum-shoulder_mas-hand_mas | 7 | 9.5 ± 0.7 | 10.4 ± 0.5 | <.001 | 1.46 |
| thigh_mas-shoulder_mas-asis_mas-psis_las-head-psis_mas-sternum | 7 | 8.4 ± 1.1 | 9.4 ± 0.9 | .02 | 0.57 |
| toe_las-psis_las-sternum-psis_mas-shoulder_las-head-asis_mas | 7 | 8.0 ± 0.9 | 9.0 ± 0.6 | <.001 | 0.62 |
| head-psis_las-asis_las-shoulder_mas-shank_las-elbow_mas-ankle_las-toe_las | 8 | 9.8 ± 1.4 | 11.1 ± 1.4 | .02 | 0.58 |
| shoulder_las-thigh_las-shank_las-wrist_mas-hand_mas-elbow_mas-ankle_las-elbow_las | 8 | 9.0 ± 0.9 | 9.8 ± 0.8 | .04 | 0.53 |
| elbow_las-shank_mas-hand_las-shank_las-thigh_las-shoulder_las-asis_mas-psis_mas | 8 | 9.1 ± 0.7 | 9.8 ± 0.7 | .02 | 0.57 |
| elbow_mas-hand_mas-wrist_mas-shank_las-thigh_las-shoulder_mas-asis_mas-psis_las | 8 | 10.8 ± 1.0 | 11.9 ± 0.9 | .02 | 0.61 |
| hand_mas-wrist_mas-ankle_las-toe_las-shoulder_mas-shank_las-elbow_mas-ankle_mas | 8 | 8.3 ± 1.1 | 9.1 ± 1.1 | .04 | 0.56 |
| asis_las-psis_mas-asis_mas-psis_las-sternum-shoulder_mas-hand_mas-wrist_mas | 8 | 11.3 ± 0.7 | 12.2 ± 0.5 | <.001 | 1.46 |
| asis_mas-psis_las-head-shoulder_mas-hand_mas-wrist_mas-shank_las-thigh_las | 8 | 10.5 ± 1.0 | 11.5 ± 0.9 | .04 | 0.54 |
| psis_mas-asis_mas-psis_las-asis_las-shoulder_mas-hand_mas-elbow_mas-thigh_las | 8 | 10.6 ± 1.0 | 11.6 ± 0.8 | .02 | 0.59 |
| thigh_las-shoulder_las-asis_mas-psis_las-sternum-psis_mas-thigh_mas-shank_mas | 8 | 10.2 ± 0.9 | 11.2 ± 0.7 | <.001 | 1.23 |
| thigh_mas-shoulder_mas-asis_mas-psis_las-head-psis_mas-shoulder_las-sternum | 8 | 9.6 ± 1.4 | 10.8 ± 1.2 | .02 | 0.61 |
| shank_las-hand_mas-elbow_mas-ankle_las-toe_las-shoulder_mas-thigh_las-shoulder_las | 8 | 8.6 ± 1.4 | 9.7 ± 1.4 | .04 | 0.54 |
| toe_las-psis_las-asis_las-asis_mas-psis_mas-shoulder_mas-thigh_las-shoulder_las | 8 | 9.4 ± 0.6 | 10.4 ± 0.6 | <.001 | 1.67 |
| head-psis_las-asis_las-shoulder_mas-shank_las-elbow_mas-ankle_las-toe_las-thigh_las | 9 | 10.6 ± 1.5 | 12.0 ± 1.4 | .02 | 0.58 |
| elbow_mas-hand_mas-wrist_mas-shank_las-thigh_las-shoulder_mas-asis_mas-psis_mas-head | 9 | 12.3 ± 1.1 | 13.5 ± 1.0 | <.001 | 0.63 |
| wrist_las-hand_las-shank_mas-elbow_las-shank_las-thigh_las-shoulder_las-asis_mas-psis_las | 9 | 10.9 ± 0.9 | 11.8 ± 0.7 | .02 | 1.11 |
| hand_mas-wrist_mas-ankle_las-toe_las-thigh_las-shoulder_mas-shank_las-elbow_mas-ankle_mas | 9 | 9.8 ± 1.2 | 10.8 ± 1.1 | .02 | 0.56 |
| asis_las-psis_mas-asis_mas-psis_las-sternum-shoulder_mas-hand_mas-wrist_mas-shank_las | 9 | 12.6 ± 1.0 | 13.8 ± 0.9 | <.001 | 0.63 |
| thigh_mas-shoulder_mas-asis_mas-psis_las-asis_las-psis_mas-head-sternum-shoulder_las | 9 | 11.9 ± 1.2 | 13.0 ± 1.1 | .02 | 0.95 |
| shank_las-hand_mas-elbow_mas-ankle_las-toe_las-shoulder_mas-thigh_las-shoulder_las-thigh_mas | 9 | 9.8 ± 1.6 | 11.1 ± 1.5 | .04 | 0.55 |
| toe_las-psis_las-asis_las-asis_mas-psis_mas-shoulder_las-sternum-shoulder_mas-thigh_las | 9 | 11.5 ± 0.9 | 12.6 ± 0.6 | <.001 | 1.42 |
| head-psis_las-asis_las-shoulder_mas-shank_las-elbow_mas-ankle_las-toe_las-thigh_las-sternum | 10 | 11.9 ± 1.6 | 13.5 ± 1.5 | .02 | 0.59 |
| wrist_las-hand_las-shank_mas-elbow_las-shank_las-thigh_las-shoulder_las-asis_mas-psis_las-asis_las | 10 | 12.6 ± 0.9 | 13.7 ± 0.8 | <.001 | 1.29 |
| hand_las-wrist_las-ankle_mas-toe_mas-shank_mas-elbow_las-shank_las-thigh_las-shoulder_las-asis_mas | 10 | 12.2 ± 1.0 | 13.1 ± 0.9 | .04 | 0.55 |
| asis_las-psis_mas-asis_mas-psis_las-sternum-shoulder_mas-hand_mas-wrist_mas-shank_las-wrist_las | 10 | 13.1 ± 0.8 | 14.1 ± 0.6 | <.001 | 1.4 |
| asis_mas-psis_las-head-shoulder_mas-hand_mas-wrist_mas-shank_las-thigh_las-elbow_las-hand_las | 10 | 12.9 ± 0.9 | 13.9 ± 0.8 | .02 | 0.6 |
| psis_las-asis_mas-psis_mas-sternum-shoulder_mas-thigh_mas-shank_mas-wrist_mas-shank_las-hand_mas | 10 | 12.6 ± 1.0 | 13.5 ± 0.7 | .04 | 0.55 |
| psis_mas-asis_mas-psis_las-asis_las-shoulder_mas-hand_mas-elbow_mas-ankle_las-elbow_las-hand_las | 10 | 12.3 ± 1.2 | 13.5 ± 0.8 | <.001 | 1.16 |
| thigh_las-shoulder_las-thigh_mas-psis_mas-sternum-psis_las-asis_las-wrist_las-hand_las-elbow_las | 10 | 12.6 ± 0.9 | 13.7 ± 0.9 | <.001 | 1.22 |
| thigh_mas-shoulder_mas-asis_mas-psis_las-asis_las-shoulder_las-thigh_las-shank_las-ankle_las-wrist_mas | 10 | 12.4 ± 1.0 | 13.8 ± 0.8 | <.001 | 1.53 |
| shank_las-hand_mas-elbow_mas-ankle_las-toe_las-shoulder_mas-thigh_las-shoulder_las-asis_las-psis_mas | 10 | 11.5 ± 1.4 | 13.1 ± 1.4 | <.001 | 0.67 |
| ankle_las-elbow_mas-hand_mas-wrist_mas-shank_las-thigh_las-shoulder_las-psis_mas-psis_las-sternum | 10 | 13.3 ± 1.4 | 14.7 ± 1.1 | .02 | 0.6 |
| toe_las-psis_las-asis_mas-shoulder_mas-thigh_las-shoulder_las-sternum-psis_mas-head-asis_las | 10 | 11.7 ± 1.3 | 13.2 ± 1.1 | <.001 | 0.68 |
| head-psis_las-asis_las-shoulder_mas-shank_las-hand_mas-wrist_mas-elbow_mas-ankle_las-toe_las-thigh_las | 11 | 14.1 ± 1.7 | 15.7 ± 1.5 | .02 | 0.6 |
| wrist_las-hand_las-shank_mas-wrist_mas-hand_mas-elbow_mas-asis_las-psis_mas-asis_mas-psis_las-sternum | 11 | 15.3 ± 1.0 | 16.1 ± 0.7 | .04 | 0.54 |
| wrist_mas-hand_mas-shank_las-elbow_mas-ankle_las-hand_las-wrist_las-asis_las-psis_las-sternum-psis_mas | 11 | 13.7 ± 1.3 | 14.9 ± 1.1 | .02 | 0.58 |
| hand_mas-wrist_mas-ankle_las-toe_las-shoulder_mas-shank_las-elbow_mas-ankle_mas-toe_mas-wrist_las-hand_las | 11 | 13.1 ± 1.6 | 14.2 ± 1.6 | .04 | 0.54 |
| asis_las-psis_mas-asis_mas-sternum-shoulder_mas-hand_mas-wrist_mas-shank_las-wrist_las-hand_las-ankle_mas | 11 | 14.6 ± 1.0 | 15.7 ± 0.9 | .02 | 0.61 |
| psis_mas-asis_mas-psis_las-asis_las-shoulder_mas-hand_mas-elbow_mas-ankle_las-elbow_las-hand_las-shank_mas | 11 | 13.7 ± 1.4 | 15.1 ± 1.1 | .02 | 0.57 |
| thigh_mas-shoulder_mas-asis_mas-psis_las-asis_las-psis_mas-head-shoulder_las-thigh_las-elbow_las-ankle_mas | 11 | 13.2 ± 1.3 | 14.5 ± 1.0 | .02 | 1.11 |
| shank_las-hand_mas-elbow_mas-ankle_las-toe_las-shoulder_mas-thigh_mas-psis_mas-asis_mas-psis_las-asis_las | 11 | 13.7 ± 1.4 | 14.9 ± 1.2 | .02 | 0.56 |
| shank_mas-elbow_las-ankle_mas-toe_mas-head-psis_las-asis_las-psis_mas-asis_mas-shoulder_las-thigh_las | 11 | 13.6 ± 1.2 | 14.9 ± 1.1 | .02 | 0.61 |
| head-psis_las-asis_las-shoulder_mas-shank_las-wrist_mas-elbow_mas-ankle_las-hand_mas-sternum-psis_mas-asis_mas | 12 | 15.3 ± 2.2 | 17.3 ± 2.1 | .02 | 0.6 |
| wrist_las-hand_las-shank_mas-wrist_mas-hand_mas-shank_las-thigh_las-shoulder_las-sternum-psis_las-asis_las-psis_mas | 12 | 16.3 ± 1.1 | 17.5 ± 0.8 | <.001 | 0.63 |
| wrist_mas-hand_mas-shank_las-elbow_mas-ankle_las-hand_las-wrist_las-asis_las-psis_las-sternum-psis_mas-asis_mas | 12 | 15.5 ± 1.3 | 16.8 ± 1.1 | .02 | 0.59 |
| hand_las-wrist_las-ankle_mas-toe_mas-shank_mas-wrist_mas-sternum-psis_mas-asis_mas-psis_las-asis_las-head | 12 | 15.9 ± 1.1 | 17.0 ± 0.8 | .02 | 0.57 |
| asis_las-psis_mas-asis_mas-psis_las-sternum-shoulder_mas-hand_mas-ankle_las-elbow_las-hand_las-wrist_las-wrist_mas | 12 | 14.8 ± 0.9 | 15.8 ± 0.7 | <.001 | 1.23 |
| thigh_las-shoulder_las-asis_mas-psis_las-asis_las-psis_mas-sternum-shoulder_mas-thigh_mas-shank_mas-hand_las-wrist_las | 12 | 16.6 ± 1.3 | 18.0 ± 0.9 | <.001 | 1.24 |
| thigh_mas-shoulder_mas-asis_mas-psis_las-asis_las-psis_mas-head-shoulder_las-thigh_las-wrist_mas-shank_las-hand_las | 12 | 14.3 ± 1.3 | 15.8 ± 1.0 | <.001 | 1.28 |
| shank_las-hand_mas-elbow_mas-ankle_las-toe_las-shoulder_mas-thigh_mas-psis_mas-asis_mas-psis_las-asis_las-shoulder_las | 12 | 15.0 ± 1.3 | 16.5 ± 1.2 | <.001 | 0.66 |
| shank_mas-elbow_las-ankle_mas-toe_mas-head-psis_las-asis_las-psis_mas-asis_mas-shoulder_las-thigh_las-shank_las | 12 | 15.3 ± 1.3 | 16.7 ± 1.2 | <.001 | 0.62 |
| head-psis_las-asis_las-shoulder_mas-shank_las-elbow_mas-ankle_las-hand_mas-wrist_mas-sternum-psis_mas-asis_mas-shoulder_las | 13 | 16.9 ± 2.1 | 19.1 ± 2.0 | .02 | 0.6 |
| sternum-psis_mas-thigh_mas-elbow_las-ankle_las-toe_las-shoulder_las-thigh_las-shank_las-hand_mas-wrist_mas-elbow_mas-ankle_mas | 13 | 14.8 ± 0.8 | 15.6 ± 0.8 | .02 | 1.0 |
| shoulder_mas-thigh_mas-elbow_las-hand_las-wrist_las-psis_las-asis_las-psis_mas-asis_mas-head-shoulder_las-thigh_las-shank_las | 13 | 17.2 ± 1.3 | 18.5 ± 1.1 | .02 | 0.59 |
| wrist_las-hand_las-shank_mas-wrist_mas-hand_mas-shank_las-thigh_las-shoulder_las-sternum-psis_las-asis_las-psis_mas-asis_mas | 13 | 18.1 ± 1.2 | 19.4 ± 0.9 | <.001 | 0.64 |
| wrist_mas-hand_mas-shank_las-elbow_mas-ankle_las-hand_las-wrist_las-asis_las-psis_las-psis_mas-asis_mas-head-toe_mas | 13 | 15.7 ± 1.4 | 16.9 ± 1.1 | .02 | 0.57 |
| hand_las-wrist_las-ankle_mas-toe_mas-shank_mas-wrist_mas-sternum-psis_mas-asis_mas-psis_las-asis_las-head-thigh_mas | 13 | 17.0 ± 1.3 | 18.3 ± 0.9 | .02 | 0.6 |
| asis_las-psis_mas-asis_mas-psis_las-sternum-shoulder_mas-hand_mas-ankle_las-elbow_las-hand_las-wrist_las-wrist_mas-shank_las | 13 | 16.1 ± 1.2 | 17.3 ± 1.0 | .02 | 0.6 |
| psis_las-asis_mas-psis_mas-sternum-shoulder_mas-hand_mas-elbow_mas-ankle_las-elbow_las-hand_las-wrist_las-asis_las-shoulder_las | 13 | 16.3 ± 1.2 | 17.8 ± 0.8 | <.001 | 1.45 |
| psis_mas-asis_mas-psis_las-asis_las-shoulder_mas-hand_mas-elbow_mas-ankle_las-elbow_las-hand_las-shank_las-wrist_mas-head | 13 | 15.0 ± 1.5 | 16.5 ± 1.0 | .02 | 0.6 |
| thigh_las-shoulder_las-asis_mas-psis_mas-sternum-psis_las-asis_las-shoulder_mas-thigh_mas-shank_mas-hand_las-wrist_las-elbow_las | 13 | 18.3 ± 1.4 | 19.9 ± 1.1 | <.001 | 0.65 |
| thigh_mas-shoulder_mas-asis_mas-psis_las-asis_las-shoulder_las-thigh_las-wrist_mas-elbow_mas-shank_las-hand_las-wrist_las-elbow_las | 13 | 16.4 ± 1.1 | 17.9 ± 1.0 | <.001 | 0.71 |
| shank_las-hand_mas-elbow_mas-ankle_las-toe_las-shoulder_mas-thigh_mas-psis_mas-asis_mas-psis_las-asis_las-sternum-shoulder_las | 13 | 16.9 ± 1.4 | 18.4 ± 1.2 | <.001 | 0.63 |
| toe_las-psis_las-sternum-psis_mas-asis_mas-shoulder_mas-thigh_mas-shank_mas-hand_las-shank_las-thigh_las-shoulder_las-ankle_mas | 13 | 15.1 ± 0.9 | 16.0 ± 0.8 | <.001 | 0.63 |
| sternum-psis_mas-thigh_mas-shank_mas-elbow_las-ankle_las-toe_las-shoulder_las-thigh_las-shank_las-hand_mas-wrist_mas-elbow_mas-ankle_mas | 14 | 16.6 ± 0.9 | 17.4 ± 0.9 | .02 | 0.89 |
| shoulder_mas-thigh_mas-shank_mas-hand_las-ankle_las-toe_las-elbow_mas-hand_mas-wrist_mas-shank_las-thigh_las-psis_mas-asis_mas-psis_las | 14 | 18.5 ± 1.5 | 19.9 ± 1.2 | .02 | 0.6 |
| elbow_mas-hand_mas-shank_las-thigh_las-shoulder_mas-asis_mas-sternum-psis_mas-head-toe_mas-ankle_mas-shank_mas-hand_las-ankle_las | 14 | 17.6 ± 1.4 | 19.1 ± 1.2 | <.001 | 0.63 |
| wrist_mas-hand_mas-shank_las-elbow_mas-ankle_las-hand_las-wrist_las-elbow_las-toe_las-shoulder_mas-sternum-psis_mas-asis_mas-head | 14 | 17.1 ± 1.4 | 18.6 ± 1.1 | <.001 | 0.63 |
| hand_mas-wrist_mas-ankle_las-toe_las-shoulder_mas-shank_las-elbow_mas-ankle_mas-toe_mas-hand_las-wrist_las-elbow_las-psis_las-asis_las | 14 | 17.6 ± 1.6 | 19.0 ± 1.5 | .02 | 0.59 |
| asis_las-psis_mas-asis_mas-psis_las-shoulder_las-thigh_las-shank_las-hand_las-wrist_las-elbow_las-ankle_las-toe_las-shoulder_mas-hand_mas | 14 | 16.9 ± 1.3 | 18.2 ± 1.2 | <.001 | 0.64 |
| psis_las-asis_mas-psis_mas-sternum-shoulder_mas-hand_mas-elbow_mas-ankle_las-elbow_las-hand_las-wrist_las-asis_las-shoulder_las-thigh_las | 14 | 17.3 ± 1.2 | 19.0 ± 0.9 | <.001 | 1.59 |
| thigh_las-shoulder_las-asis_mas-psis_mas-sternum-psis_las-asis_las-shoulder_mas-thigh_mas-shank_mas-hand_las-wrist_las-elbow_las-ankle_las | 14 | 18.7 ± 1.4 | 20.3 ± 1.1 | <.001 | 1.26 |
| thigh_mas-shoulder_mas-asis_mas-psis_las-asis_las-psis_mas-head-shoulder_las-thigh_las-wrist_mas-shank_las-hand_las-wrist_las-elbow_las | 14 | 17.9 ± 1.4 | 19.5 ± 1.1 | <.001 | 0.62 |
| shank_mas-elbow_las-ankle_mas-toe_mas-elbow_mas-ankle_las-wrist_mas-shank_las-thigh_las-shoulder_las-psis_mas-asis_mas-psis_las-asis_las | 14 | 17.5 ± 2.3 | 19.4 ± 2.2 | .02 | 0.57 |
| ankle_las-elbow_mas-hand_mas-wrist_mas-shank_las-thigh_las-shoulder_las-asis_mas-psis_mas-sternum-psis_las-asis_las-shoulder_mas-thigh_mas | 14 | 19.2 ± 1.7 | 21.2 ± 1.3 | <.001 | 0.67 |
| head-psis_las-asis_las-shoulder_mas-shank_las-elbow_mas-ankle_las-hand_mas-wrist_mas-sternum-psis_mas-asis_mas-shoulder_las-thigh_las-elbow_las | 15 | 18.7 ± 1.9 | 20.9 ± 1.7 | <.001 | 0.65 |
| sternum-psis_mas-head-toe_mas-ankle_mas-hand_mas-wrist_mas-elbow_mas-ankle_las-toe_las-shoulder_las-thigh_las-shank_las-wrist_las-elbow_las | 15 | 17.5 ± 1.1 | 18.5 ± 1.1 | .02 | 0.56 |
| shoulder_las-thigh_las-shank_las-hand_mas-elbow_mas-ankle_mas-elbow_las-ankle_las-toe_las-asis_las-psis_mas-asis_mas-shoulder_mas-thigh_mas-shank_mas | 15 | 17.4 ± 1.1 | 18.7 ± 1.1 | .02 | 1.18 |
| shoulder_mas-thigh_mas-elbow_las-ankle_las-toe_las-asis_las-psis_mas-asis_mas-psis_las-sternum-shoulder_las-thigh_las-shank_las-elbow_mas-hand_mas | 15 | 19.0 ± 1.2 | 20.4 ± 1.0 | <.001 | 0.61 |
| elbow_mas-hand_mas-shank_las-thigh_las-shoulder_mas-asis_mas-psis_mas-head-toe_mas-ankle_mas-shank_mas-hand_las-ankle_las-toe_las-asis_las | 15 | 18.4 ± 1.5 | 19.7 ± 1.3 | .04 | 0.53 |
| wrist_las-hand_las-shank_mas-elbow_las-shank_las-elbow_mas-ankle_las-hand_mas-wrist_mas-asis_mas-psis_mas-sternum-psis_las-asis_las-shoulder_mas | 15 | 19.9 ± 2.2 | 21.7 ± 2.3 | .04 | 0.55 |
| wrist_mas-hand_mas-shank_las-elbow_mas-ankle_las-hand_las-wrist_las-elbow_las-shank_mas-thigh_mas-shoulder_mas-sternum-psis_mas-asis_mas-head | 15 | 20.1 ± 1.9 | 21.8 ± 1.7 | .04 | 0.54 |
| asis_las-psis_mas-asis_mas-psis_las-shoulder_las-thigh_las-shank_las-hand_las-wrist_las-elbow_las-ankle_las-toe_las-shoulder_mas-hand_mas-wrist_mas | 15 | 18.8 ± 1.3 | 20.2 ± 1.2 | <.001 | 0.65 |
| psis_las-asis_mas-psis_mas-sternum-shoulder_mas-hand_mas-elbow_mas-ankle_las-wrist_las-elbow_las-hand_las-shank_mas-thigh_mas-head-shoulder_las | 15 | 20.1 ± 1.7 | 21.7 ± 1.2 | .02 | 0.59 |
| thigh_las-shoulder_las-asis_mas-psis_mas-sternum-psis_las-asis_las-shoulder_mas-thigh_mas-shank_mas-hand_las-wrist_las-elbow_las-ankle_mas-ankle_las | 15 | 19.9 ± 1.6 | 21.7 ± 1.4 | <.001 | 0.61 |
| shank_mas-elbow_las-ankle_mas-toe_mas-elbow_mas-hand_las-wrist_las-shank_las-hand_mas-wrist_mas-psis_mas-asis_mas-psis_las-asis_las-shoulder_las | 15 | 17.8 ± 1.2 | 19.0 ± 1.0 | .04 | 0.53 |
| ankle_mas-hand_las-wrist_las-shank_mas-elbow_las-thigh_mas-psis_mas-asis_mas-psis_las-asis_las-shoulder_las-thigh_las-shank_las-elbow_mas-ankle_las | 15 | 20.1 ± 2.8 | 22.0 ± 3.0 | .02 | 0.56 |
| head-psis_las-asis_las-shoulder_mas-shank_las-elbow_mas-ankle_las-hand_mas-wrist_mas-sternum-psis_mas-asis_mas-shoulder_las-thigh_las-elbow_las-hand_las | 16 | 20.3 ± 1.9 | 22.7 ± 1.7 | <.001 | 0.66 |
| shoulder_mas-thigh_mas-elbow_las-ankle_las-toe_las-asis_las-psis_mas-asis_mas-psis_las-sternum-shoulder_las-thigh_las-shank_las-hand_mas-elbow_mas-wrist_las | 16 | 19.2 ± 1.2 | 20.5 ± 1.0 | .02 | 0.58 |
| elbow_mas-hand_mas-shank_las-thigh_las-shoulder_mas-asis_mas-psis_mas-head-toe_mas-ankle_mas-elbow_las-hand_las-ankle_las-toe_las-asis_las-psis_las | 16 | 20.1 ± 1.5 | 21.7 ± 1.3 | .02 | 0.6 |
| wrist_las-hand_las-shank_mas-elbow_las-shank_las-hand_mas-elbow_mas-ankle_las-wrist_mas-asis_mas-psis_mas-sternum-psis_las-asis_las-shoulder_mas-thigh_mas | 16 | 20.6 ± 2.4 | 22.5 ± 2.3 | .02 | 0.56 |
| wrist_mas-hand_mas-shank_las-elbow_mas-ankle_las-toe_las-shoulder_mas-psis_las-asis_las-shoulder_las-thigh_las-psis_mas-asis_mas-head-toe_mas-ankle_mas | 16 | 20.1 ± 1.9 | 22.3 ± 1.9 | <.001 | 0.66 |
| hand_mas-wrist_mas-ankle_las-ankle_mas-toe_mas-shoulder_mas-shank_las-elbow_mas-shank_mas-hand_las-wrist_las-elbow_las-toe_las-head-shoulder_las-thigh_las | 16 | 17.3 ± 1.2 | 18.4 ± 1.1 | .02 | 0.59 |
| asis_las-psis_mas-asis_mas-psis_las-shoulder_las-thigh_las-shoulder_mas-hand_mas-wrist_mas-shank_las-hand_las-wrist_las-elbow_mas-ankle_las-elbow_las-ankle_mas | 16 | 18.1 ± 1.2 | 19.7 ± 1.0 | <.001 | 0.75 |
| psis_las-asis_mas-psis_mas-sternum-shoulder_mas-hand_mas-elbow_mas-ankle_las-wrist_las-elbow_las-hand_las-shank_mas-thigh_mas-head-shoulder_las-thigh_las | 16 | 21.1 ± 1.6 | 22.9 ± 1.3 | <.001 | 0.64 |
| psis_mas-asis_mas-psis_las-asis_las-shoulder_mas-hand_mas-elbow_mas-ankle_las-wrist_las-elbow_las-hand_las-shank_mas-thigh_mas-head-shoulder_las-ankle_mas | 16 | 20.5 ± 1.9 | 22.3 ± 1.4 | .04 | 0.55 |
| thigh_las-shoulder_las-asis_mas-psis_mas-sternum-psis_las-asis_las-shoulder_mas-thigh_mas-shank_mas-hand_las-wrist_las-elbow_las-ankle_mas-ankle_las-toe_las | 16 | 21.7 ± 1.7 | 23.5 ± 1.4 | .02 | 0.61 |
| thigh_mas-shoulder_mas-asis_mas-psis_las-asis_las-shoulder_las-thigh_las-wrist_mas-shank_las-elbow_mas-ankle_las-hand_mas-ankle_mas-toe_mas-wrist_las-hand_las | 16 | 19.6 ± 2.4 | 21.9 ± 2.3 | .02 | 0.59 |
| shank_mas-elbow_las-ankle_mas-toe_mas-elbow_mas-ankle_las-wrist_mas-shank_las-hand_mas-psis_mas-asis_mas-psis_las-asis_las-shoulder_las-thigh_las-shoulder_mas | 16 | 19.2 ± 2.9 | 21.6 ± 3.1 | .02 | 0.56 |
| ankle_las-elbow_mas-hand_mas-wrist_mas-shank_las-thigh_las-shoulder_las-asis_mas-psis_mas-sternum-psis_las-asis_las-head-toe_mas-wrist_las-elbow_las | 16 | 21.5 ± 2.0 | 23.6 ± 1.8 | <.001 | 0.64 |
| toe_las-psis_las-sternum-psis_mas-asis_mas-shoulder_mas-thigh_las-shoulder_las-ankle_mas-shank_mas-hand_las-shank_las-elbow_mas-hand_mas-wrist_mas-asis_las | 16 | 19.3 ± 1.5 | 20.7 ± 1.4 | .04 | 0.54 |
| head-psis_las-asis_las-shoulder_mas-shank_las-elbow_mas-hand_mas-wrist_mas-sternum-psis_mas-asis_mas-shoulder_las-thigh_las-elbow_las-ankle_las-toe_las-hand_las | 17 | 20.9 ± 1.5 | 22.7 ± 1.2 | <.001 | 1.32 |
| shoulder_las-thigh_las-shoulder_mas-thigh_mas-shank_mas-hand_las-wrist_las-ankle_mas-elbow_las-ankle_las-elbow_mas-shank_las-hand_mas-wrist_mas-psis_mas-asis_mas-psis_las | 17 | 21.0 ± 2.8 | 23.0 ± 2.9 | .04 | 0.54 |
| wrist_mas-hand_mas-shank_las-elbow_mas-ankle_las-toe_las-shoulder_mas-psis_las-asis_las-shoulder_las-thigh_las-psis_mas-asis_mas-head-toe_mas-ankle_mas-shank_mas | 17 | 21.5 ± 2.0 | 23.7 ± 1.9 | <.001 | 0.62 |
| hand_mas-wrist_mas-ankle_las-toe_las-shoulder_mas-shank_las-elbow_mas-ankle_mas-toe_mas-shank_mas-hand_las-wrist_las-elbow_las-shoulder_las-thigh_las-psis_mas-asis_mas | 17 | 21.7 ± 1.9 | 23.4 ± 1.8 | .02 | 0.59 |
| asis_las-psis_mas-asis_mas-psis_las-shoulder_las-thigh_las-shoulder_mas-hand_mas-wrist_mas-shank_las-elbow_mas-ankle_las-wrist_las-hand_las-elbow_las-ankle_mas-toe_mas | 17 | 22.2 ± 2.1 | 24.5 ± 2.0 | <.001 | 0.62 |
| thigh_mas-shoulder_mas-asis_mas-psis_las-asis_las-psis_mas-head-shoulder_las-thigh_las-wrist_mas-shank_las-elbow_mas-ankle_las-hand_mas-ankle_mas-toe_mas-wrist_las | 17 | 21.0 ± 2.6 | 23.4 ± 2.4 | .02 | 0.57 |
| shank_mas-elbow_las-ankle_mas-toe_mas-elbow_mas-ankle_las-wrist_mas-shank_las-hand_mas-psis_mas-asis_mas-psis_las-asis_las-shoulder_las-thigh_las-shoulder_mas-thigh_mas | 17 | 20.2 ± 2.8 | 22.6 ± 2.9 | .02 | 0.56 |
| ankle_las-elbow_mas-hand_mas-wrist_mas-shank_las-thigh_las-shoulder_las-asis_mas-psis_mas-sternum-psis_las-asis_las-head-toe_mas-wrist_las-hand_las-elbow_las | 17 | 23.2 ± 2.1 | 25.5 ± 1.8 | <.001 | 0.64 |
| toe_las-psis_las-sternum-psis_mas-asis_mas-shoulder_mas-thigh_las-shoulder_las-ankle_mas-toe_mas-wrist_las-shank_las-elbow_mas-hand_mas-wrist_mas-asis_las-elbow_las | 17 | 20.4 ± 1.3 | 21.9 ± 1.2 | <.001 | 0.64 |
| toe_mas-ankle_mas-elbow_las-shank_mas-hand_las-ankle_las-shank_las-elbow_mas-wrist_mas-hand_mas-head-shoulder_mas-psis_mas-asis_mas-psis_las-asis_las-shoulder_las | 17 | 23.0 ± 2.0 | 24.7 ± 2.0 | .04 | 0.54 |
| head-psis_las-asis_las-shoulder_mas-shank_las-elbow_mas-hand_mas-wrist_mas-sternum-psis_mas-asis_mas-shoulder_las-thigh_las-elbow_las-ankle_las-toe_las-hand_las-wrist_las | 18 | 22.7 ± 1.5 | 24.6 ± 1.3 | <.001 | 1.35 |
| wrist_las-hand_las-shank_mas-elbow_las-ankle_las-hand_mas-elbow_mas-ankle_mas-toe_mas-shoulder_las-sternum-psis_las-asis_las-psis_mas-asis_mas-head-shoulder_mas-thigh_mas | 18 | 22.9 ± 1.5 | 24.5 ± 1.2 | <.001 | 1.17 |
| thigh_mas-shoulder_mas-asis_mas-psis_las-asis_las-psis_mas-head-shoulder_las-thigh_las-elbow_las-ankle_las-wrist_mas-shank_las-elbow_mas-ankle_mas-toe_mas-wrist_las-hand_las-sternum | 19 | 22.2 ± 1.7 | 24.2 ± 1.3 | <.001 | 1.31 |
| asis_las-psis_mas-asis_mas-psis_las-shoulder_las-thigh_las-shoulder_mas-hand_mas-wrist_mas-shank_mas-hand_las-wrist_las-elbow_mas-ankle_las-elbow_las-ankle_mas-toe_mas-shank_las-sternum-head | 20 | 22.9 ± 1.5 | 24.7 ± 1.2 | <.001 | 1.32 |
| ML direction | | | | | |
| shoulder_las-thigh_las | 2 | 0.6 ± 0.2 | 0.9 ± 0.2 | <.001 | 1.5 |
| shoulder_mas-thigh_mas | 2 | 0.6 ± 0.2 | 0.9 ± 0.2 | <.001 | 1.5 |
| thigh_las-shoulder_las-sternum | 3 | 2.1 ± 0.2 | 2.5 ± 0.3 | <.001 | 1.59 |
| thigh_mas-shoulder_mas-asis_mas | 3 | 1.5 ± 0.4 | 2.1 ± 0.4 | <.001 | 1.5 |
| hand_mas-wrist_mas-ankle_las-toe_las-shoulder_mas-thigh_las | 6 | 4.1 ± 0.4 | 4.5 ± 0.4 | .04 | 0.54 |
| elbow_las-shank_mas-hand_las-shank_las-thigh_las-shoulder_las-asis_mas | 7 | 4.7 ± 0.6 | 5.5 ± 0.8 | <.001 | 1.14 |
| elbow_las-shank_mas-hand_las-shank_las-thigh_las-shoulder_las-asis_mas-psis_mas | 8 | 5.5 ± 0.7 | 6.4 ± 1.0 | .02 | 1.06 |
| elbow_las-shank_mas-hand_las-shank_las-wrist_las-ankle_mas-toe_mas-thigh_las-shoulder_las | 9 | 5.1 ± 0.6 | 5.9 ± 0.9 | .04 | 1.06 |
| wrist_las-hand_las-shank_mas-elbow_las-shank_las-thigh_las-shoulder_las-asis_mas-psis_las | 9 | 6.7 ± 0.8 | 7.7 ± 1.0 | <.001 | 1.11 |
| hand_mas-wrist_mas-ankle_las-toe_las-thigh_las-shoulder_mas-shank_las-elbow_mas-ankle_mas | 9 | 5.6 ± 0.6 | 6.4 ± 0.7 | <.001 | 1.23 |
| hand_las-wrist_las-ankle_mas-toe_mas-shank_mas-elbow_las-shank_las-thigh_las-shoulder_las-asis_mas | 10 | 7.7 ± 0.7 | 8.6 ± 1.0 | .02 | 1.06 |
| thigh_mas-shoulder_mas-asis_mas-psis_las-asis_las-shoulder_las-thigh_las-shank_las-ankle_las-wrist_mas | 10 | 8.0 ± 1.1 | 9.1 ± 1.2 | .04 | 0.96 |
| hand_mas-wrist_mas-ankle_las-ankle_mas-toe_mas-shoulder_mas-shank_las-elbow_mas-shank_mas-hand_las-wrist_las-elbow_las-toe_las-head-shoulder_las-thigh_las | 16 | 11.5 ± 1.2 | 12.7 ± 1.2 | .04 | 1.0 |
| thigh_mas-shoulder_mas-asis_mas-psis_las-asis_las-shoulder_las-thigh_las-wrist_mas-shank_las-elbow_mas-ankle_las-hand_mas-ankle_mas-toe_mas-wrist_las-hand_las | 16 | 11.2 ± 1.4 | 12.9 ± 2.0 | .02 | 1.0 |
| ankle_mas-hand_las-ankle_las-shank_las-hand_mas-wrist_mas-shank_mas-elbow_las-thigh_mas-psis_mas-asis_mas-wrist_las-psis_las-asis_las-shoulder_mas-sternum-shoulder_las-thigh_las-head | 19 | 15.9 ± 1.8 | 18.0 ± 2.3 | .02 | 1.03 |
| shank_mas-elbow_las-ankle_las-wrist_mas-shank_las-hand_mas-elbow_mas-ankle_mas-toe_mas-wrist_las-hand_las-psis_mas-asis_mas-psis_las-asis_las-shoulder_las-thigh_las-shoulder_mas-thigh_mas-head | 20 | 13.7 ± 1.6 | 15.9 ± 2.6 | .02 | 1.04 |
| V direction – no significant differences | | | | | |
| **Slow walking speed** | | | | | |
| AP direction – no significant differences | | | | | |
| ML direction | | | | | |
| shoulder_las-thigh_las | 2 | 0.6 ± 0.2 | 0.9 ± 0.3 | .02 | 1.17 |
| V direction – no significant differences | | | | | |

S-VII. Average step length and variability (expressed as standard deviation) of the step length in PD and control groups.

Supplementary Table 3. Average step length [m] in three gait speeds

| Gait speed | PwPD | Controls |
| --- | --- | --- |
| Fast | .68 ± .14^*^ | .78 ± .13 |
| Preferred | .59 ± .11^**^ | .7 ± .1 |
| Slow | .49 ± .11 | .53 ± .09 |

PwPD -people with Parkinson’s Disease. * - p<.05; ** - p<.01; *** - p<.001

Supplementary Table 4. Average step length [m] in three gait speeds

| Gait speed | PwPD | Controls |
| --- | --- | --- |
| Fast | .07 ± .04^*^ | .04 ±. 02 |
| Preferred | .08 ± .04^***^ | .04 ± .02 |
| Slow | .06 ± .02^**^ | .04 ± .03 |

PwPD -people with Parkinson’s Disease. * - p<.05; ** - p<.01; *** - p<.001
